# Neither injury induced macrophages within the nerve, nor the environment created by Wallerian degeneration is necessary for enhanced in vivo axon regeneration after peripheral nerve injury

**DOI:** 10.1186/s12974-024-03132-5

**Published:** 2024-05-27

**Authors:** Aaron D. Talsma, Jon P. Niemi, Richard E. Zigmond

**Affiliations:** Department of Neurosciences, Case Western Reserve University, 10900 Euclid Avenue, Cleveland, OH 44106-4975 USA

**Keywords:** Arginase 1, Axotomy, CCR2, Clodronate, Conditioning lesion, Dorsal root, Macrophages, Neuroimmune, Regeneration, Zymosan

## Abstract

**Background:**

Since the 1990s, evidence has accumulated that macrophages promote peripheral nerve regeneration and are required for enhancing regeneration in the conditioning lesion (CL) response. After a sciatic nerve injury, macrophages accumulate in the injury site, the nerve distal to that site, and the axotomized dorsal root ganglia (DRGs). In the peripheral nervous system, as in other tissues, the macrophage response is derived from both resident macrophages and recruited monocyte-derived macrophages (MDMs). Unresolved questions are: at which sites do macrophages enhance nerve regeneration, and is a particular population needed.

**Methods:**

*Ccr2* knock-out (KO) and *Ccr2*^*gfp/gfp*^ knock-in/KO mice were used to prevent MDM recruitment. Using these strains in a sciatic CL paradigm, we examined the necessity of MDMs and residents for CL-enhanced regeneration in vivo and characterized injury-induced nerve inflammation. CL paradigm variants, including the addition of pharmacological macrophage depletion methods, tested the role of various macrophage populations in initiating or sustaining the CL response. In vivo regeneration, measured from bilateral proximal test lesions (TLs) after 2 d, and macrophages were quantified by immunofluorescent staining.

**Results:**

Peripheral CL-enhanced regeneration was equivalent between crush and transection CLs and was sustained for 28 days in both *Ccr2* KO and WT mice despite MDM depletion. Similarly, the central CL response measured in dorsal roots was unchanged in *Ccr2* KO mice. Macrophages at both the TL and CL, but not between them, stained for the pro-regenerative marker, arginase 1. TL macrophages were primarily CCR2-dependent MDMs and nearly absent in *Ccr2* KO and *Ccr2*^*gfp/gfp*^ KO mice. However, there were only slightly fewer Arg1^+^ macrophages in CCR2 null CLs than controls due to resident macrophage compensation. Zymosan injection into an intact WT sciatic nerve recruited Arg1^+^ macrophages but did not enhance regeneration. Finally, clodronate injection into *Ccr2*^*gfp*^ KO CLs dramatically reduced CL macrophages. Combined with the *Ccr2*^*gfp*^ KO background, depleting MDMs and TL macrophages, and a transection CL, physically removing the distal nerve environment, nearly all macrophages in the nerve were removed, yet CL-enhanced regeneration was not impaired.

**Conclusions:**

Macrophages in the sciatic nerve are neither necessary nor sufficient to produce a CL response.

**Supplementary Information:**

The online version contains supplementary material available at 10.1186/s12974-024-03132-5.

## Introduction

Peripheral nerves are well known for their ability to regenerate, often for weeks [1]. A peripheral nerve injury also primes axotomized neurons to regenerate faster after a subsequent injury in a phenomenon called the conditioning lesion (CL) response. Additionally, the CL response permits growth through the restrictive CNS environment, observed in the central branch of pseudounipolar sensory neurons conditioned by peripheral axotomy [2–6]. Maximal regeneration enhancement is achieved 8 days after the first nerve injury regardless of the number of CLs given [7], implying that processes induced by an injury and not the injury itself are responsible for the CL response. Injury induces a transcriptional program in neurons, which is necessary for enhanced regeneration [8–10]. A component of the CL response is neuron intrinsic as enhanced regeneration and regeneration-associated gene (RAG) expression can be induced in sensory neuron monocultures [8]. However, this response lasts less than 3 days, implying there are other critical components in vivo [11]. Importantly, injuring the central branch of sensory neurons does not induce RAGs or enhance regeneration, indicating a peripheral factor, such as inflammation, is necessary for the CL response [4, 12–15].

A sciatic nerve injury creates 4 to 5 distinct compartments, depending on the type of injury, where inflammation and local signaling could influence axonal regeneration. Those compartments include the dorsal root ganglia (DRGs), the nerve segments proximal and distal to the lesion, and the lesion site, which in a transection injury is split into proximal and distal nerve (DN) stumps. These macrophages are suggested to promote axon growth but an unresolved question is which populations are required [16–18]. After a single injury, macrophages have many roles in the repair and regeneration process. In the DN, macrophages assist with myelin clearance [19, 20], growth factor secretion [21, 22], angiogenesis [21, 23, 24], and efferocytosis [17], which may promote regeneration and the CL response [21, 25, 26]. Retrograde signals in injured axons are necessary for enhancing regeneration [27, 28]. In particular, retrograde transport of activated signal transducer and activator of transcription 3 (STAT3) is necessary for accumulation of phospho-STAT3 in the nucleus and CL-enhanced regeneration [29, 30]. Phosphorylation of STAT3 at two sites downstream of gp130 and neurotrophin signaling is necessary for the maximal axonal outgrowth [31]. Macrophages can produce ligands for both receptor classes suggesting a possible mechanism by which nerve macrophages enhance regeneration.

Both resident and injury-induced macrophages in the nerve and DRG were initially thought to be predominantly bone marrow derived (i.e., monocyte derived macrophages; MDMs) [26, 32]. Accordingly, loss of either CCR2 or CCL2 was reported to dramatically impair DN and DRG macrophage accumulation [18, 19, 33, 34] and CL-enhanced regeneration in vitro [18, 33], suggesting MDMs and DRG macrophages are necessary for the CL response. However, recent studies demonstrated that the DRG macrophage response is dominated by proliferation of a true resident population [16, 17] independent of CCR2 signaling [35]. The nerve response is dominated by MDMs and thus is impaired in *Ccr2* null animals [17, 19, 36], although resident compensation results in only a 50% macrophage reduction [35]. Given these new results, the impaired CL response reported in *Ccr2* KOs may be due to either the overall decrease in nerve macrophages or an ineffective phenotype adopted by the residents due to their distinct lineage. Indeed, the transition of injury-induced nerve macrophages from a Ly6C^hi^ to Ly6C^lo^ phenotype may be necessary for CL enhanced regeneration in the dorsal columns [17].

In this study, we sought to examine the role of CCR2 signaling and injury-induced nerve macrophages in CL-enhanced regeneration in vivo. We used Ccr2 KO animals and *Ccr2*^*gfp*^ knockin/knockout (KI/KO) animals [37], which express GFP under the control of the endogenous *Ccr2* locus and can distinguish MDMs from resident macrophages responding to nerve injury [35, 38], to examine both the role of MDMs in regeneration and the source and phenotype of nerve macrophages. Using a variety of CL assays, we determined that myelin removal, CCR2 signaling, DN MDMs, and the DN environment were not necessary to initiate or sustain CL-enhanced regeneration in vivo. We find lesion site macrophages are a distinct population that express the pro-regenerative marker Arg1 independent of their lineage and CCR2 signaling. However, when they are depleted with the other injury-induced nerve macrophages, we observe no effect on CL-enhanced regeneration in vivo.

## Materials and methods

### Mice

Eight- to 20 week-old age matched mice were used for all studies. It has previously been shown that there is no sexual dimorphism in the CL response [35]; therefore, males and females were used in approximately equal numbers for all groups in all experiments. Mice were housed under 12 h:12 h light: dark cycle with *ad libitum* access to food (LabDiet; Prolab RHM 3000) and water. The strains in this study were acquired from Jackson Laboratory (Bar Harbor, ME, USA) and maintained in our animal facility. C57BL/6J mice were used as WT animals (Jackson Laboratory; #000664) and as controls for *Ccr2* KOs (Jackson Laboratory; #0004999). *Ccr2*^*gfp/gfp*^ KI/KO animals (Jackson Laboratory; #027619) were bred with C57BL/6J to create heterozygous *Ccr2*^*gfp/+*^ controls or with themselves to produce *Ccr2*^*gfp/gfp*^, which served as a second KO strain for CCR2.

### Surgeries

All surgical procedures were approved by the Case Western Reserve University Institutional Animal Care and Use Committee. Surgeries were performed under isoflurane anesthesia. After all surgeries, animals were given bupivacaine subcutaneously at the incision and carprofen subcutaneously at the scruff. Wound clips were used to close the wound and were removed 14 d after surgery, if applicable.

*Peripheral regeneration and the response to a CL.* A small skin incision was made in the mid-thigh on the right and left hind limb. On the right side, the sciatic nerve was exposed and transected just before the trifurcation producing a CL, and an approximately 2 mm forceps-width of nerve was removed distally. With the intrinsic retraction of the nerves, this created a critical nerve gap (greater than 3 mm) which was not bridged in our experiments [39]. On the left side, the trifurcation was exposed producing a sham control nerve. In one experiment, rather than transecting the right sciatic nerve, it was crushed just before the trifurcation. Wounds were closed with a wound clip, and the mice were allowed to recover for the “conditioning period” which was 7, 14 or 28 d as indicated. Then, the original incisions were reopened, and the sciatic nerves were exposed at the level of the greater trochanter of the femur. The sciatic nerves were crushed bilaterally using ultrafine hemostats (Fine Science Tools, Forest City, CA, USA; 13006-12) for 45 s just distal to the greater trochanter producing the TL. The wounds were closed again with wound clips. The mice recovered for 2 or 5 d as indicated and then were sacrificed by CO_2_ inhalation. The sciatic nerves were removed, pinned in a 35 mm petri dish with dental wax on the bottom so the nerves were straight, and fixed overnight in 4% paraformaldehyde in PBS. Growth of axons in the left sciatic nerve was taken as control (unconditioned) growth.

*CL and dorsal root regeneration.* First, a unilateral sciatic nerve transection was performed as described above. After a 7 d conditioning period, the TL was made on the L4 dorsal roots. First, an expanded L4 laminectomy was made. The L5 spinous process space is in line with the iliac crest and was identified by that landmark [40]. An incision extending from ∼L6-L3 was made, and the spinal muscles were detached with sharp tipped 5 mm micro scissors (Fine Science Tools (FST; 15003-08) from the L3, L4, and L5 vertebrae to completely expose the L4 DRG. Then, using fine-tipped rongeurs (FST; 16221-14), the L5 spinous process, which projects slightly anteriorly, was removed to expose the L4-L5 intervertebral space. Next, we removed the L4 laminae, transverse processes, and a portion of the L4 pedicles to expose the L4 nerve roots, the L5 superior articular processes to visualize the L4 DRG, and a caudal portion of the L3 laminae to give more access to the L4 dorsal root. Within this surgical window, the L4 dorsal and ventral roots are the most lateral and can be seen entering the L4 DRG sitting at the caudal border of the L4 vertebral body. Sterile Lactated Ringer’s solution (VEDCO, St. Joseph, MO, USA; 50989-883-17) was used to irrigate the surgical site as needed. Using fine micro scissors (FST; 15000-08), the meninges were carefully opened by making a midline sagittal incision followed by lateral incisions starting from the rostral end of the first incision. The meninges were folded back to expose the L4 dorsal roots. Each was crushed for 15 s with an angled suture tying forceps that we filed slightly to ensure the faces contacted completely (FST; 11063-07). The ventral root was usually crushed at the same time since separating it from the dorsal root was likely to cause collateral tissue damage. The meningeal flaps were then moved back into place, and the window was covered with BIOBRANE (Smith + Nephew, Inc., Andover, MA, USA). The remaining muscles were placed back near their original position on top of the BIOBRANE, and the wound was closed. The mice were allowed 3 d to recover before they were sacrificed, and the L4 DRGs with spinal nerve and nerve roots were collected, pinned, and fixed as with the sciatic nerves.

### Zymosan conditioning of the sciatic nerve

Zymosan is a yeast cell wall extract, which can stimulate inflammation in the nervous system [41–43]. We used zymosan to induce inflammation in the sciatic nerve in an attempt to condition the nerve. Zymosan A (Millipore Sigma. St. Louis, MO, USA; Z4250) was suspended in sterile PBS at a concentration of 5 µg/µL for injection. As zymosan does not dissolve, the mixture was agitated before each injection to resuspend it. Animals were anesthetized, and the sciatic nerve was exposed bilaterally, as described above. On the right side, a 10 µL Nanofil syringe (World Precision Instruments, Sarasota, FL, USA) with a 35 G beveled needle (WPI; NF35BV-2) was inserted near the trifurcation and moved along the axis of the nerve until the needle tip was at least ∼5 mm into the nerve. The nerves were slowly injected with 0.2 µL of either PBS or 5 µg/µL zymosan (1 µg total) while the needle was pulled out to spread the injection along the length of the nerve. Animals recovered for 7 d to allow for inflammation and conditioning to occur. Then bilateral crush TLs were made, and 2 d later the animals were sacrificed. The nerves were treated as described for the CL paradigm.

### Conditioning lesion with CL site treatments

Treatment of CL macrophages with an M1 stimulation mixture, an arginase inhibitor, or clodronate liposomes was given by nerve injection. First, a unilateral sciatic nerve transection was performed as described above with a 7 d conditioning period. Then, on the day of the transection and every day after until the animals were sacrificed, 1 µL of treatment was injected into the cut end of the nerve using a 10 µL Nanofil syringe (WPI) and a 35 G beveled needle (WPI). To ensure the treatment and any damage caused by the injection was restricted to the CL area, the needle was inserted just past the end of the bevel, which is ∼250 μm. If the sciatic nerve had segregated into two fascicles before the trifurcation, each fascicle was injected with a portion of the 1 µL. After the 7 d conditioning period, proximal TLs were made, and 2 d were allowed for regeneration before the animals were sacrificed and nerves collected as described above.

*M1 stimulation injections*: Lipopolysaccharide (LPS) stimulates the classic M1 macrophage phenotype in vitro [44] and stimulates an inflammatory environment in the CNS [45, 46]. M1 and M2 polarization signals activate antagonistic transcriptional programs, and many M2 signals function through signal transducer and activator of transcription 6 (STAT6) activation [47]. Thus, we used LPS (Sigma-Aldrich; O55:B5 E. coli, L5418-2ML) and the STAT6 inhibitor AS1517499 (Millipore Sigma; SML1906) to promote M1 polarization in vivo. In vitro, M1 and M2 phenotypes fade in the absence of polarizing signals [48] so we treated the CL sites every day. According to the manufacturer, the IC50 of AS1517499 is 21 nM in vitro. We used a 50X concentration (10 µM) to maintain this dose locally. LPS was delivered at a concentration of 0.5 µg/µL or 0.5 µg per day. To prepare the M1 stimulation cocktail, AS1517499 was first prepared as a 10 mM stock solution in DMSO. An aliquot was serially diluted 1:10 and then 1:50 in sterile PBS to make a 20 µM solution. The LPS was received in a 1 µg/µL aqueous solution, which was mixed 1:1 with the 20 µM STAT6 inhibitor solution to make the 10 µM STAT6 inhibitor, 0.5 µg/µL LPS cocktail for injection. Vehicle solution was 50 µL RNase free water, 0.1 µL DMSO and 49.9 µL sterile PBS. A total of 9 injections were given during a standard in vivo CL paradigm as described above.

*Arginase inhibitor injections*: The Arg1 inhibitor, alpha-amino acid N(omega)-hydroxy-nor-l-arginine (nor-NOHA; Cayman Chemical, Ann Arbor, MI, USA; 10,006,861), was dissolved in DMSO to create a 100 mM stock solution (25 mg in 843 µL DMSO). The stock solution was diluted 1:10 in PBS to create a working solution of 10 mM. PBS and DMSO were mixed 1:10 to create the solution used for the vehicle controls. A total of 9 injections were given during a standard in vivo CL paradigm as described above.

*Clodronate injections*: Clodronate acts intracellularly to induce apoptosis, and encapsulating it in liposomes targets its uptake and toxicity to macrophages [49]. 1 µL of clodronate liposomes (FormuMax, Sunnyvale, CA, USA; Clophosome-A, F70101C-A) or control liposomes (FormuMax; F70101-A) were injected into the nerve at the CL. For WT animals, the CL was injected daily for a total of nine injections during a standard in vivo CL paradigm as described above. For *Ccr2*^*gfp*^KOs, the CL was injected the day of the transection, the day after, and then every other day for a total of 5 injections.

### Luxol fast blue (LFB) for myelin visualization

LFB staining was performed on 20 μm sections of the distal sciatic nerve. Slides were rehydrated in water for 5 min, moved to 35% ethanol for 5 min, and then to 70% ethanol for 5 min before being placed in sealed slide containers with filtered 0.1% LFB solution (Electron Microscopy Sciences; 26681-01). The slides were incubated at 60–65 °C overnight, rinsed briefly by dipping first in 95% ethanol and then in distilled water, destained by incubating in 0.05% lithium carbonate (w/v) in ddH_2_0 for 30 s, and then immediately quenched by rinsing and incubating in 70% ethanol for 5 min. Slides were dehydrated by incubating in 95% ethanol and then 100% ethanol for 5 min each, soaked in xylenes for 5 min, and then cover-slipped with VectaMount Mounting Medium (Vector Laboratories, Newark, CA, USA; H-5000). Slides were imaged at 20x with transmitted light on a Zeiss AxioImager M2 microscope with the investigator blinded to the experimental groups.

### Immunofluorescence and imaging

Pairs of sham and manipulated sciatic nerves from each animal were embedded in Tissue-Tek O.C.T. compound (Electron Microscopy Sciences), sectioned at 10, 20 and 40 μm using a cryostat (Leica Biosystems, Wetzlar, Germany), and direct mounted onto Superfrost Plus slides (Thermo Fisher; 12-550-15). Tissue was stained on the slides in humidified chambers, using a PAP pen ring to contain the staining solutions. All tissue was rehydrated for 20 min in 0.25% PBS-Triton X100 (PBS-TX). Blocking buffer was 10% normal donkey serum in either PBS-TX or 0.25% PBS-Tween20 (PBS-T). Sections were incubated overnight at 4 °C with primary antibodies and subsequently incubated at room temperature in secondary antibodies for either 1 h (for 10 μm sections) or 2 h (for 20 and 40 μm sections). Tissues were washed with PBS-TX or PBS-T 4 times after the primary and secondary antibody incubation steps for 10 min each for 10 and 20 μm sections and 15 min for 40 μm sections. The last two washes after the secondary antibody step were done with PBS. Primary antibodies combinations diluted in PBS-TX blocking buffer were as follows: rabbit anti-SCG10 (Novus Biologicals, Centennial, CO, USA; NBP1-49461) at 1:4000; rabbit anti-SCG10 at 1:4000 and rat anti-CD68 (Bio-Rad Laboratories, Hercules, CA, USA; clone FA-11, MCA1957) at 1:400; rat anti-CD68 at 1:400 and chicken anti-Arg1 [50; Aves Labs; #1146] at 1:5000; sheep anti-GFP (Bio-Rad; 4745 − 1051) at 1:500, rat anti-CD68 at 1:400 and chicken anti-Arg1 at 1:5000; sheep anti-GFP at 1:500 and rat anti-CD68 at 1:400. Rabbit anti-GFP (ThermoFisher; A-11,122) at 1:800 and rat anti-F4/80 at 1:1000; rat anti-CD68 at 1:400, Rabbit anti-iNOS (Sigma; N7782) at 1:500, and chicken anti-Arg1 at 1:5000 were diluted in PBS-T blocking buffer. When staining for F4/80, tissue was incubated in preheated 10 mM sodium citrate buffer, pH 6.0, for 20 min at 95 °C after rehydration for antigen retrieval. The following secondary antibodies were diluted 1:400 in the same blocking buffer as the primary antibodies: AF488 donkey anti-rabbit (Jackson ImmunoResearch; 711-545-152), AF488 donkey Fab_2_ anti-rat (Jackson ImmunoResearch; 712-546-150), AF488 donkey Fab_2_ anti-chicken (Jackson ImmunoResearch; 703-546-155), AF488 donkey Fab_2_ anti-sheep (Jackson ImmunoResearch; 713-546-147), AF594 donkey anti-rat (Jackson ImmunoResearch; 712-585-153), AF594 donkey anti-chicken (Jackson ImmunoResearch; 703-585-155), AF647 donkey Fab_2_ anti-rabbit (Jackson ImmunoResearch; 711-606-152), AF647 donkey Fab_2_ anti-rat (Jackson ImmunoResearch; 712-606-150), AF647 donkey Fab_2_ anti-chicken (Jackson ImmunoResearch; 703-606-155). 4′,6-Diamidino-2-phenylindole (DAPI) 1:1000 in PBS (ThermoFisher; D1306) was used to label cell nuclei. Vectamount PLUS (Vector Laboratories; H-1900) was used to mount 40 μm sections and Fluoro-Gel (Electron Microscopy Sciences; 17985-10) was used to mount 10 and 20 μm sections.

A Zeiss AxioImager M2 wide field microscope was used to take 3-color images (DAPI, AF488 and AF647) of 10 μm sections. Images to quantify nerve regeneration and macrophages in 40 μm sections, and 4-color images (DAPI, AF594, AF488 and AF647) were taken using a Zeiss LSM 800. All slides stained in the same batch (i.e., with the same blocking buffer and antibody solutions) for an experiment were imaged with constant settings (e.g., laser powers, scan speed, resolution, PMT voltages, and exposure times). All confocal stacks were run through a 3 by 3 median filter to remove noise before they were maximally projected for quantification. The investigator was blinded to the groups before quantification. Controls without primary antibody were used to guide selection of minimum values for positive staining when doing cell counts or measuring percent area stained. Images were quantified using ImageJ software (1.53).

### In vivo CL regeneration analyses

In vivo regeneration analysis was performed using FIJI and excel macros as previously described [35]. Briefly, the center of the crush site was marked, and images were blinded using the excel macro. In the FIJI macro, a 500 μm wide region of interest (ROI) was placed on the crush site, a threshold for positive staining was set, and the upper and lower bounds of the nerve were traced for all nerves. The excel macro then calculated the fraction of regenerating axons at 100 μm intervals from the crush site, and the average axon length in each nerve based on the number of pixels at each distance and at the crush site. The dorsal root crush sites violated assumption 2 of the analysis from Talsma et al. [35], and thus the FIJI macro was modified. A 300 μm wide ROI was placed on the intact proximal portion of the crush and was used to represent the maximum number of axons. The distal crush was often notably disrupted, accounting in part for reduced regeneration, and was defined by a second ROI and excluded from the analysis. The threshold was set, and the roots were traced as for the sciatic nerves. The same formulas were used to calculate the fraction of regenerating axons and average axon length.

### Statistics and experimental design

Statistical tests were performed in Prism 10 (GraphPad, San Diego, CA). All bar graphs show the mean +/- SEM. Means were compared using a Two-Way ANOVA with Sidak’s multiple comparisons post hoc tests unless otherwise noted, α = 0.05. Using this test, only comparisons within genotypes or treatments and within injury conditions were made. Other comparisons are not meaningful and were not performed (e.g. a sham WT group was not compared to a conditioned KO group). For each experiment, approximately equal numbers of male and female mice were used. At least 3 animals of each sex were slated for an experimental group when planning, depending on the availability of the genotype, and 4 of each sex was considered ideal in case a sample was lost. If a cage slated for an experiment contained extra mice those mice were added to their corresponding experimental group. Each animal was considered to be an experimental unit. Therefore, the minimum n for each experiment is 4 to 10, and the n for each sex is 2 to 5.

## Results

### Ccr2 KO animals have normal enhancement of peripheral regeneration after a peripheral CL

**Fig. 1:**
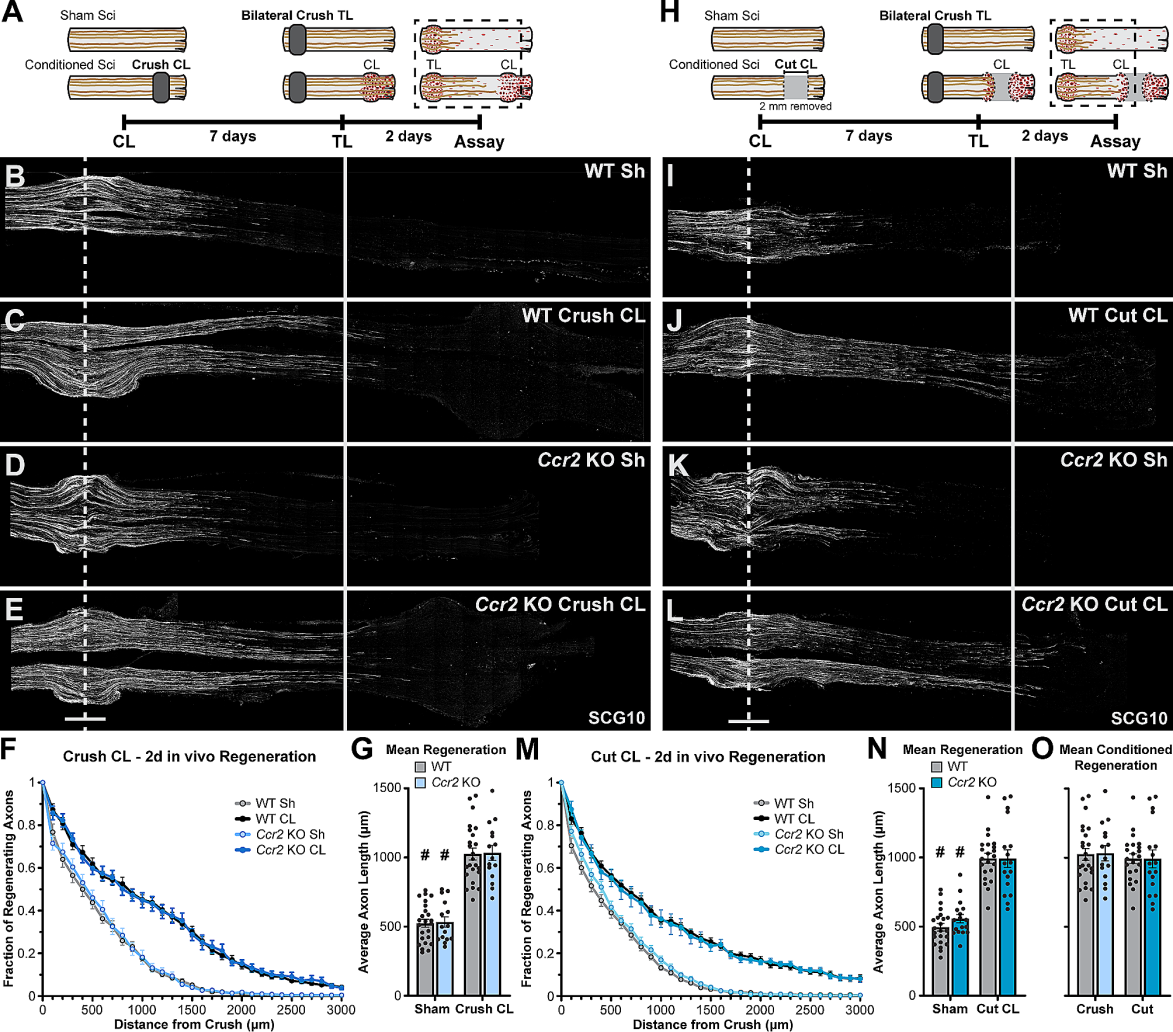
Sciatic nerves of *Ccr2* KO animals show normal regeneration after a single crush injury and CL-enhanced regeneration. WT and *Ccr2* KO animals underwent an in vivo CL assay: a unilateral distal sciatic nerve injury, either a crush or transection, followed by a 7 d conditioning period, then bilateral proximal crush test lesions followed by 2 d for regeneration. **A, H.** Diagram showing the time course and relative positions of the CL and TL performed on sciatic nerves for the in vivo Crush CL (A) or Cut CL (H) assays, and the dotted box indicates the tissue area examined for the assays. **B-E and I-L.** Representative images of regenerating nerves from WT and *Ccr2* KO mice immunostained for SCG10, which labels regenerating sensory axons, in 40 μm sections. **F, M.** Axon regeneration quantified at 100 μm intervals as the fraction of regenerating axons relative to the crush site for WT and *Ccr2* KO mice after a Crush CL (F) and a Cut CL (H). **G, N, O.** Mean regeneration distance calculated by integrating SCG10 immunofluorescent staining of regenerating axons for WT and *Ccr2* KO mice after a Crush CL (G) and a Cut CL (N). **O.** Comparison of Crush and Cut conditioned regeneration distance. Unconditioned regeneration (Sh; B, D, I, K), represents the baseline growth rate. Conditioned regeneration (CL; C, E, J, L) was the same between genotypes and type of CL, and significantly increased compared to contralateral unconditioned nerves. The dotted line indicates the center of the crush site which was considered to be 500 μm wide, and the solid line is 3000 μm from the crush. Scale bar = 500 μm. # indicates a significant (*p* < 0.05) difference between the Sh (unconditioned) and CL (conditioned) regeneration within a genotype. *N* = 24 WT Crush, 22 WT Cut, 15 *Ccr2* KO Crush, 17 *Ccr2* KO Cut

To measure CL-enhanced regeneration in vivo, we developed a peripheral CL assay, which generally is a unilateral CL of the sciatic nerve at the trifurcation (usually a transection) followed by a conditioning period (usually 7 d), and then bilateral proximal crush lesions to test the regenerative ability of the nerves (i.e., test lesion, TL) followed by a regeneration period (usually 2 d). Using variants of this assay, we examined the effect of reducing injury-induced macrophage recruitment using both the *Ccr2* KO mouse [18, 19] and the *Ccr2*^*gfp/gfp*^ mouse (referred to henceforth as *Ccr2*^*gfp*^ KO). CCR2-dependent macrophage recruitment was hypothesized to be required for CL-enhanced regeneration based on the impairment in neurite outgrowth observed in cultured ganglia from *Ccr2* KO mice [18]. Since *Ccr2* null animals are primarily deficient in both total macrophages (50% reduction) and MDMs specifically (80–90% reduction) in the DN [35], it suggests these macrophages are necessary for CL-enhanced regeneration. This was first tested by performing crush CLs in WT and *Ccr2* KOs (Fig. 1A). Since crushed axons grow beyond the injury within 2 days [35, 51], this paradigm allows the crush-conditioned axons to interact with the DN environment during the 7 d conditioning period. The *Ccr2* KOs show normal regeneration enhancement indicating that the DN MDMs are not necessary for the CL response (Fig. 1B-G). To further test the necessity of DN macrophages, we performed a transection CL assay in which an approximately 2 mm nerve segment distal to the transection is removed to prevent reattachment of the proximal and distal stumps [39] and thus any interaction of the injured axons with the DN environment (Fig. 1H). This paradigm was used to test regeneration enhancement in both *Ccr2* KOs compared to WT controls (Fig. 1I-N) and in *Ccr2*^*gfp*^ KO mice compared to *Ccr2*^*gfp/+*^ heterozygous controls (referred to henceforth as *Ccr2*^*gfp*^ het; Fig. S1 A-E). Surprisingly, CL-enhanced regeneration in *Ccr2* KOs and *Ccr2*^*gfp*^ KOs was significantly increased compared to baseline regeneration in sham conditioned nerves and indistinguishable from CL-enhanced regeneration in their respective controls (Fig. 1I-N and Fig S1). Importantly, there was no difference in CL-enhanced regeneration between the crush and transection CL groups in WT and *Ccr2* KOs (Fig. 1O) showing that loss of the DN environment in the transection CL paradigm does not impair CL-enhanced regeneration. Together, this suggests that neither CCR2-recruited MDMs nor the DN environment, including resident and recruited macrophages, are necessary for enhancing regeneration over a 7 d conditioning period.

The CL response in DRG neuronal cultures fades within 3 d of plating [11], indicating that a key function of the in vivo milieu, and reportedly the presence of activated macrophages [52], is to sustain the CL response. Thus, perhaps CCR2 signaling is necessary to recruit and replenish macrophages to sustain the inflammatory response and maintain CL-enhanced regeneration during the weeks required for functional recovery. To test this, TLs were performed 14 or 28 d after a transection CL in WT and *Ccr2* KOs (Fig. S2 A). Interestingly, CL-enhanced regeneration was maintained for 28 d, and the enhancement was as robust after 14 or 28 d as after the 7 d CL in both genotypes (Fig. S2 B-H). This indicates that CCR2 signaling, CCR2-dependent MDMs, and the DN environment are not necessary for maintaining the CL response.

The most well studied role of macrophages in the injured sciatic nerve is their clearance of myelin debris during Wallerian degeneration (WD) [53, 54]. Incomplete myelin clearance is thought to be inhibitory to regenerating axons [21, 55, 56], and significant myelin clearance distal to an injury site begins at 3 d after an injury [19, 57]. Thus, it was unlikely that myelin was cleared during the 2 d regeneration period in the region immediately distal to the TLs, however faster myelin clearance from conditioned nerves could promote enhanced regeneration. Luxol fast blue (LFB) staining was used to label myelin in cut-conditioned WT and *Ccr2* KO nerves given a 7, 14, or 28 d conditioning period and the contralateral sham nerves that only received a crush TL. The region immediately distal to the TL was quantified in both sham and conditioned nerves (Fig. S3 N). The presence of myelin, as measured by the percent area stained by LFB, was unchanged by genotype or the presence of a CL in the 7 and 14 d conditioning groups (Fig. S3 A-H, M). In the 28 d conditioning group, there was a small but significant decrease in the amount of myelin staining in WT and *Ccr2* KO CL groups compared to the contralateral nerves (Fig. S3 I-M). However, approximately 80% of the nerve area was still stained with LFB in all groups, consistent with previously reported LFB values in naïve nerves [19, 21], indicating a lack of degeneration. The region quantified in Fig. S3 (Fig. S3 N) corresponds to the regenerating segment quantified in Fig. 1I-N and S2, demonstrating that cut-conditioned axons readily regenerate into an undegenerated nerve.

### Recruited macrophages and CCR2 signaling are not required for enhancing regeneration in centrally projecting axons

**Fig. 2:**
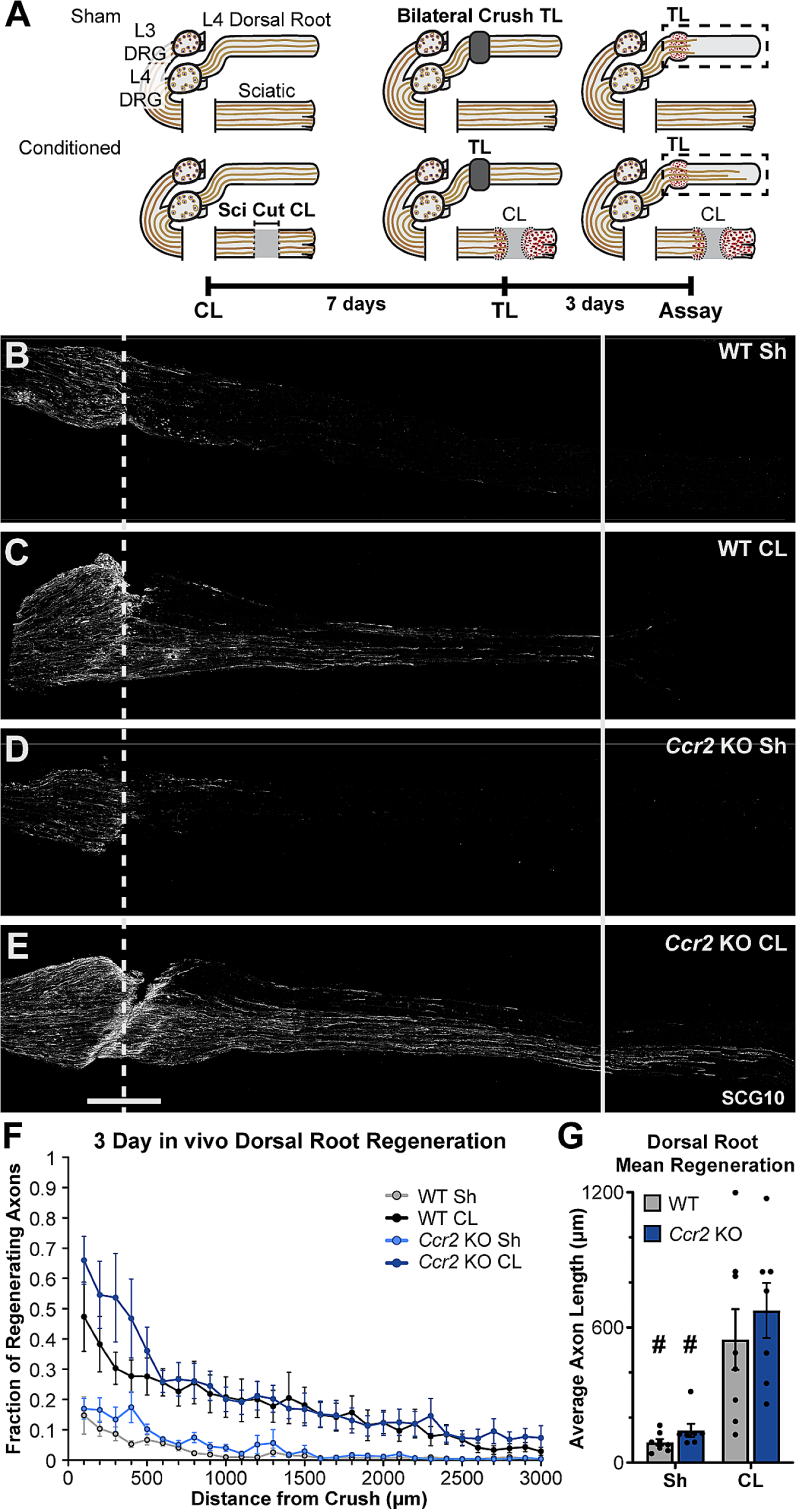
The central process of sensory neurons shows enhanced regeneration after a peripheral CL in *Ccr2* KO animals. **A.** Diagram showing the sciatic CL, dorsal root TLs and the time course used for the in vivo dorsal root regeneration assay performed on WT and *Ccr2* KO animals. **B-E.** Representative images of regenerating dorsal roots immunostained for SCG10 in 40 μm sections. Unconditioned regeneration (B, D) shows that the poor intrinsic regenerative capacity of dorsal root axons is the same in both genotypes. Conditioned regeneration (CL; C, F) was also the same between genotypes and significantly increased compared to contralateral unconditioned roots. The dotted line indicates the center of the crush site which was considered to be 500 μm wide, and the solid line is 3000 μm from the crush. Scale bar = 500 μm. **F.** Axon regeneration quantified at 100 μm intervals as the fraction of regenerating axons relative to the axons in the 300 μm proximal to the crush site. The fraction of axons at each distance from the crush was calculated from ratios of SCG10 immunofluorescence. **G.** Mean regeneration distance calculated by integrating SCG10 immunofluorescent staining of regenerating axons. # indicates a significant (*p* < 0.05) difference between the Sh (unconditioned) and CL (conditioned) regeneration within a genotype. *N* = 7–8 per group

At baseline, the peripheral branch of sensory neurons has a greater growth capacity than the central branch, but both can be conditioned after a peripheral nerve injury [5, 58, 59]. Some early studies on the CL response suggested that the peripheral and central CL responses differ in that a peripheral nerve transection, but not a crush, enhances dorsal root and dorsal column regeneration [5, 60], whereas either lesion enhances peripheral nerve regeneration [e.g., 3, 61]. In addition, one of the first papers implicating macrophages in regeneration, found that injecting macrophages directly into the DRG only enhanced dorsal root regeneration [62]. Thus, we hypothesized that MDMs may only be necessary for enhancing regeneration of the central branch of sensory neurons. This was tested in WT and *Ccr2* KO mice using a transection CL paradigm with bilateral L4 dorsal root crush TLs and a 3 d regeneration period (Fig. 2A). There was dramatic regeneration enhancement in conditioned dorsal root axons in *Ccr2* KOs which was indistinguishable from that in WT mice (Fig. 2B-G), demonstrating CCR2 signaling, CCR2-dependent MDMs, and the DN environment are dispensable for enhancing central regeneration.

### Macrophages at lesion sites are a spatially restricted population of arginase 1^+^ cells

Our transection CL paradigm combined with the use of CCR2 null animals suggests that MDMs and the environment distal to the transection (i.e., the distal stump and DN) do not play a role in CL-enhanced regeneration. Thus, we examined the macrophages that remained in our transection CL paradigm, which are primarily lesion site macrophages found in the TL and CL. Additionally, crush lesions (i.e., our TLs) cause a unique WD-independent inflammation and macrophage recruitment [63], and recent work has indicated that there are spatial differences in gene and protein expression between the immune cells responding to a nerve injury site and those responding to the DN to participate in WD [64]. It is not known if other types of lesions also induce a distinct macrophage phenotype, the source of those macrophages, or if they play a role in regeneration enhancement. We sought to investigate these questions.

**Fig. 3:**
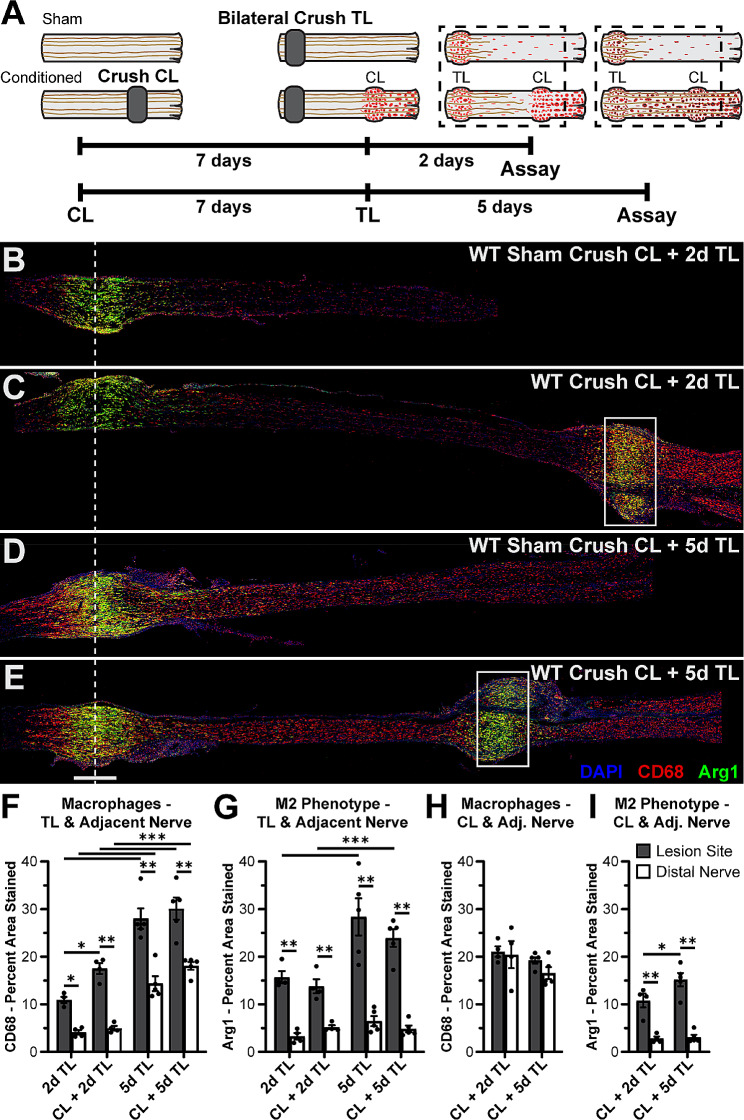
Arginase 1 expression is primarily associated with macrophages within crush sites. **A.** Diagram showing WT animals given a unilateral 7 d crush CL, followed by crush TLs with a 2 d or 5 d regeneration period. The dotted box indicates the area examined in the assays. The “Sham” contralateral nerve only received the TL. **B-C.** Representative sciatic nerves from animals given a crush CL and a 2 d TL, immunostained for the macrophage marker CD68 and the proregenerative macrophage marker arginase 1 (Arg1). **D-E.** Representative sciatic nerves from animals given a crush CL and a 5 d TL, immunostained for CD68 and Arg1. For B-E, the dotted line indicates the center of the crush TL which was nominally 600 μm wide, and the box is 600 μm wide and placed at the crush CL. **F.** Macrophages at and immediately distal to the TL site represented by CD68 percent area stained. CD68 was measured for all groups by placing a 600 μm wide rectangular ROI centered over the TL site (Lesion Site) and then a second 600 μm wide ROI adjacent to the distal edge of the first ROI (Distal Nerve). ROIs were adjusted for the height of the nerve. **G.** M2 phenotype in macrophages at and immediately distal to the TL site represented by Arg1 percent area stained. Arg1 was measured for all groups as in F. **H.** Macrophages at and immediately distal to the CL site represented by CD68 percent area stained. CD68 was measured for all groups as in F except the first ROI was placed over the CL site. **I.** M2 phenotype in macrophages at and immediately distal to the CL site represented by Arg1 percent area stained. Arg1 was measured for all groups as in H. Scale bar = 500 μm. * *p* < 0.05; ** *p* < 0.01; *** *p* < 0.001. *N* = 4–5 per group

Macrophages can take on a spectrum of activation states that include the classically activated pro-inflammatory phenotype (M1) or the alternatively activated anti-inflammatory phenotype (M2) [65, 66]. M2 macrophages have been posited to be beneficial for axonal regeneration and can be labeled by staining for arginase 1 (Arg1) [67, 68]. To examine lesion site macrophages without the lesion site complexity present after a transection injury, an experiment was performed using crush CLs and TLs because they create spatially restricted lesion sites that are visually apparent in the tissue, and whose boundaries can be defined by the instrument used to create them. Animals received a unilateral crush CL followed by a 7 d conditioning period and then bilateral crush TLs with either a 2 d or a 5 d regeneration period (Fig. 3A). Macrophages were labeled by CD68 staining, which strongly labels all macrophages but can be expressed at lower levels in other myeloid populations [69–71]. Nerves were stained for DAPI, Arg1, and CD68 and were quantified using 600 μm wide regions of interest because the lesions were made using a hemostat with a nominal width of 600 μm. Arg1^+^ CD68^+^ macrophages were present specifically in the crushed areas under all injury conditions and as early as 2 d after a single TL (Fig. 3B-E). These lesion site macrophages are distinct from the Arg1^−^ CD68^+^ macrophages located distal to all injury sites which presumably participate in WD, evidenced by the consistently low Arg1 expression in the nerve immediately distal to the lesions (Fig. 3G, I) regardless of the presence of macrophages (Fig. 3F, H). Thus, the lesion-associated macrophages are distinct from those in other areas of the injured nerve, which could indicate that they have a special function after injury, perhaps in axon regeneration.

### Origins of Lesion Site macrophages in *Ccr2*^*gfp*^ mice

**Fig. 4:**
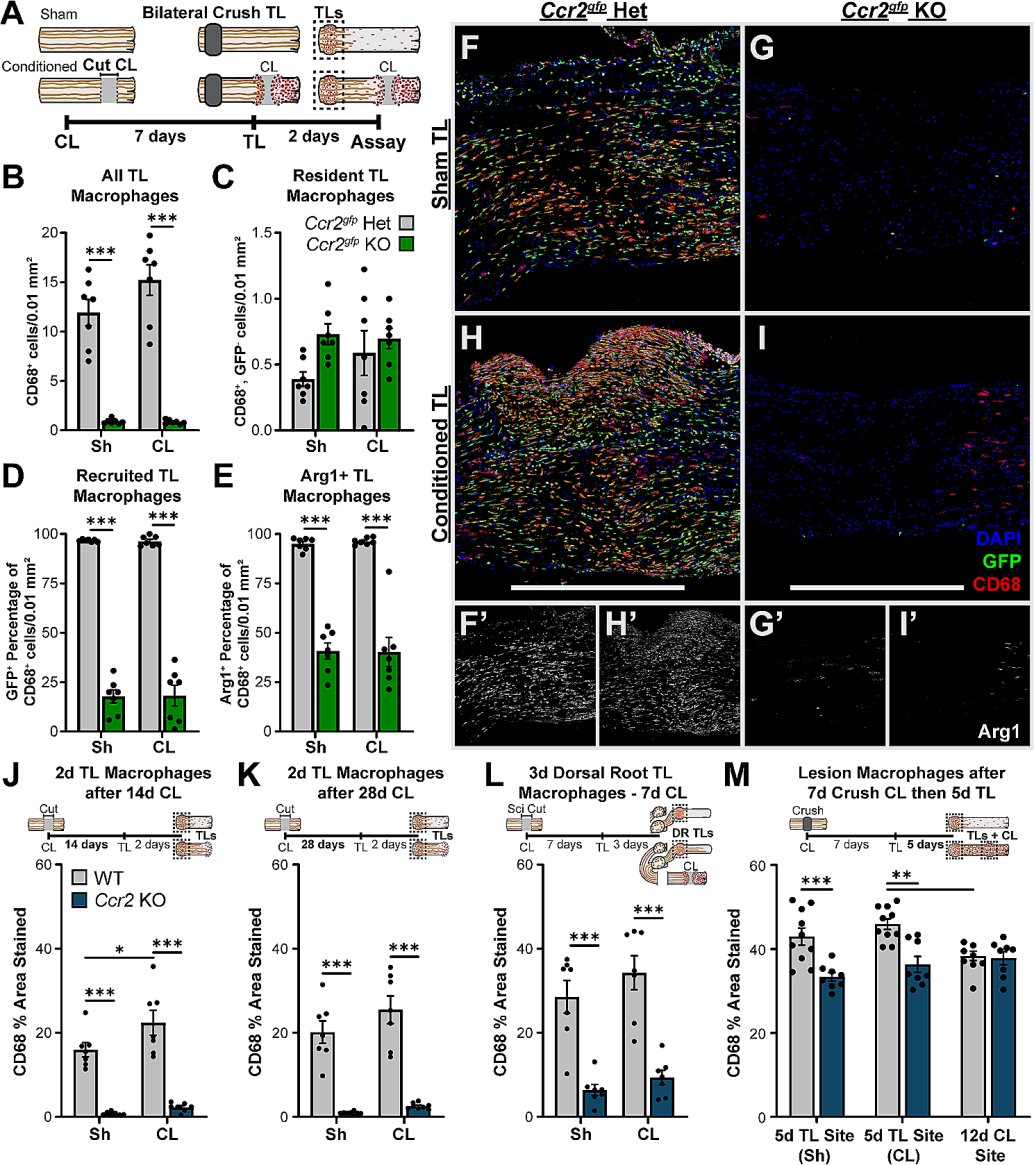
TL site macrophages are primarily CCR2^+^ MDMs and are rapidly recruited to control but not *Ccr2* null TL sites. **A.** Diagram showing the lesion paradigm with the dotted boxes indicating the location quantified in B-I. **B-E.** Quantification of the macrophages responding to the *Ccr2*^*gfp*^ het and KO TLs shown in F-I. Ten micron sections were immunostained with CD68 to label all macrophages, GFP to label infiltrating MDMs, and Arg1 to label proregenerative macrophages and cells were counted in a 600 µm wide by 300 µm tall area centered on the TL. **B.** The total macrophages in the TL sites is represented by the number of CD68^+^ cells per 0.01 mm^2^ and is significantly higher in both *Ccr2*^*gfp*^ het TL sites compared to the KOs. **C.** Resident macrophages quantified as CD68^+^GFP^−^ cells per 0.01 mm^2^. **D.** Recruited macrophages quantified by percentage of CD68^+^GFP^+^ cells per 0.01 mm^2^. Nearly all TL site macrophages are derived from CCR2^+^ infiltrating monocytes and are absent in the 2 d TL of *Ccr2*^*gfp*^ KOs. **E.** The percentage of CD68^+^ macrophages per 0.01 mm^2^ that express Arg1 are significantly decreased in *Ccr2*^*gfp*^ KOs. **F-I.** Representative images of macrophages in the TL sites of *Ccr2*^*gfp*^ het and KO animals from a CL paradigm. **F’-I’.** Insets of Arg1 staining in images in A-D. **J-M. ***Ccr2* KOs also have greatly diminished numbers of macrophages in their TLs compared to WT, quantified by the percent area stained of CD68 in the TL site of 40 μm sections. Macrophages are significantly reduced from *Ccr2* KO TLs in both the 14 d CL paradigm (I) and the 28 d CL paradigm (J) as well as dorsal root TL 3 d after injury (K). **L.** Quantification of TL sites in the 7 d CL with a 5 d TL injury paradigm shows that the macrophage deficiency is largely temporary. Scale bar = 600 μm. ** *p* < 0.01; *** *p* < 0.001. *N* = 7–10 per group

The *Ccr2*^*gfp*^ animals were next used to examine the source and phenotype of macrophages found at the TL and CL sites in control and *Ccr2* null backgrounds. We first analyzed the TLs of the nerves from the transection CL paradigm in Figure S1 (Fig. 4A). In the *Ccr2*^*gfp*^ hets, macrophages were recruited to the TL sites in approximately equal numbers in unconditioned and conditioned nerves; however, in the *Ccr2*^*gfp*^ KOs, macrophages were nearly completely absent from both TL sites (Fig. 4B-I). Approximately 95% of macrophages at the TL site in the heterozygous controls were GFP^+^ indicating that they are recruited MDMs (Fig. 4D). In the DN of *Ccr2*^*gfp*^ KOs, resident macrophages proliferate, compensating partially for the lack of MDMs [35], but the scarcity of TL macrophages indicates residents are not able to compensate in a lesion 2 d post injury. In the *Ccr2*^*gfp*^ hets, 90% of TL macrophages express Arg1 by 2 d post injury (Fig. 4E). Macrophages are also greatly reduced in the TL sites of all *Ccr2* KO nerves (Fig. 4J-M). However, the macrophage deficiency in the KOs decreases markedly with time, as demonstrated by comparing WT and *Ccr2* KO nerves in a crush CL paradigm with a 7 d conditioning and 5 d regeneration period. Macrophages are only slightly reduced in the *Ccr2* KOs at 5 d post-TL and are equivalent in the 12 d crush CL as measured by CD68% area stained in 40 μm sections (Fig. 4M). Together, these data indicate CCR2 is required for rapid recruitment of macrophages to nerve lesion sites, but alternative sources of macrophages, either proliferating residents or non-CCR2 recruited MDMs, can eventually compensate in the absence of CCR2. Notably, this compensation also occurs more rapidly than in the DN [19, 35], suggesting that additional or more effective signals stimulating proliferation or CCR2-independent recruitment are activated at the lesion site. Indeed, in nerve crush injuries on mice with mutations preventing axon degeneration, macrophage recruitment to the DN, but not the crush site, is impaired [63, 64]. These data also demonstrate that TL macrophages are neither necessary nor sufficient for CL-enhanced regeneration since TL macrophages are present in the WT sham CL nerves, which do not have enhanced regeneration, and absent in the CCR2 null conditioned nerves, which have enhanced regeneration (Fig. 4B-I J-K, and 4 L correspond to nerves in Fig. S1, S2, and 2 respectively).

**Fig. 5:**
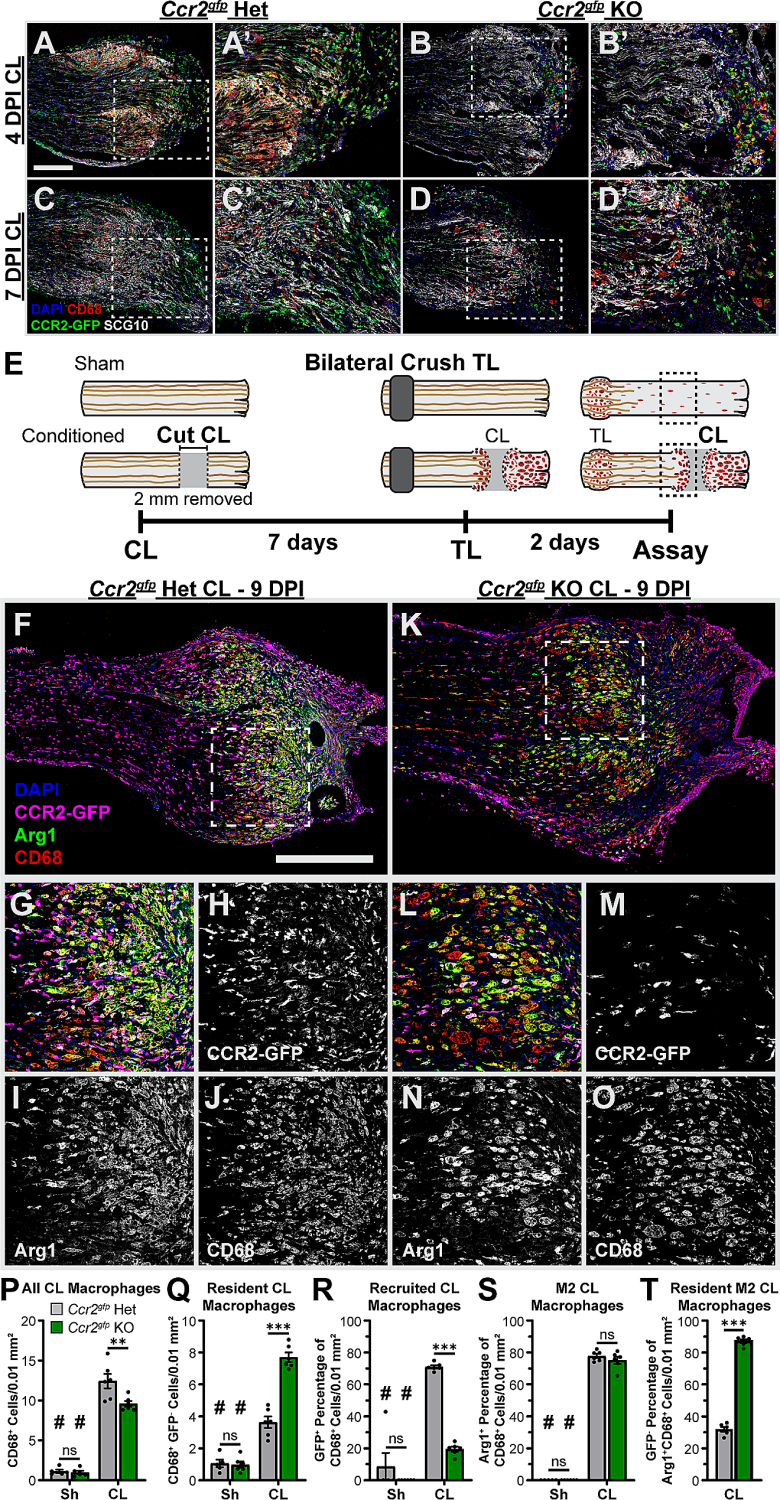
Cut axons reside in the CL with macrophages during the conditioning period, and Arg1^+^ CL site macrophages can be derived from either CCR2^+^ recruited macrophages or CCR2^−^ resident macrophages. **A-D.** Representative images of the proximal CL 4 and 7 days post-injury (DPI) of *Ccr2*^*gf*p^ hets and KOs, showing axons residing near CL macrophages during the conditioning period. Ten micron sections were immunostained for SCG10 labeling cut and regenerating axons, CD68 labeling all macrophages, and GFP labeling recruited macrophages. Dotted box indicates the area displayed in A’-D’. Scale bar = 250 μm. **A’-D’.** Enlarged 500 by 600 μm images from A-D. **E.** Diagram showing the lesion paradigm with the dotted boxes indicating the location examined in F-S. **F-O.** Representative images of *Ccr2*^*gf*p^ het and KO proximal CLs after the in vivo regeneration paradigm. **F, K.** Ten micron sections immunostained for GFP, labeling recruited CCR2^+^ macrophages shown in magenta, CD68, labeling all macrophages shown in red, and Arg1, labeling proregenerative macrophages shown in green. With these colors, recruited Arg1^+^CD68^+^GFP^+^ macrophages appear white and resident Arg1^+^CD68^+^GFP^−^ macrophages appear yellow. Arg1^−^ resident (CD68^+^ GFP^−^) and recruited (CD68^+^ GFP^+^) appear red and magenta, respectively. Dotted boxes indicate the magnified images in G-H and L-O. Scale bar = 500 μm. **G-J.** Enlarged portion of the *Ccr2*^*gfp*^ het CL site in F illustrates that most macrophages are Arg1^+^CCR2^+^ recruited macrophages although a few are resident derived. H-J Show single grayscale channels of G. **L-O.** Enlarged portion of the *Ccr2*^*gfp*^ KO CL site in K illustrates macrophages are mostly resident derived (CD68^+^CCR2^−^ cells) but are still Arg1^+^. M-O Show single channel grayscale images from L. **P-S.** Quantification of the macrophages in the CL sites shown in F-O by counting cells in an approximately 250 μm square centered on the inflammation at the cut nerve end in CLs or 3 mm distal to the TL in Sh. **P.** Total macrophages in the CL site, quantified as the number of CD68^+^ cells per 0.01 mm^2^, is significantly higher in *Ccr2*^*gfp*^ hets compared to KOs. **Q.** Resident macrophages, quantified as the number of GFP^−^CD68^+^ cells per 0.01 mm^2^, shows a significant increase in residents in *Ccr2*^*gfp*^ KO CLs compared to *Ccr2*^*gfp*^ hets. **R.** Recruited macrophages quantified as the GFP^+^ percentage of CD68^+^ cells per 0.01 mm^2^. Most CL macrophages are GFP^+^ in *Ccr2*^*gfp*^ hets, indicating they are derived from CCR2^+^ infiltrating monocytes, and are reduced but not absent in *Ccr2*^*gfp*^ KOs. **S.** M2 macrophages, quantified as the Arg1^+^ percentage of CD68^+^ cells per 0.01 mm^2^, demonstrates similar proportions of M2 CL macrophages in both genotypes. **T.** Proportion of M2 macrophages derived from residents, quantified as the GFP^−^ percentage of Arg1^+^CD68^+^ cells per 0.01 mm^2^, reveals residents expand and adopt the appropriate phenotype when MDMs are deficient in *Ccr2*^*gfp*^ KOs. ** *p* < 0.01. *** *p* < 0.001. # indicates a significant (*p* < 0.05) difference between the Sh (unconditioned) and CL (conditioned) groups within a genotype. *N* = 6–7 per group

Having excluded a role for TL macrophages in enhancing regeneration, we turned to the CL macrophages in our transection paradigm (Fig. 1H). Because this paradigm prevents reattachment of the proximal and distal stumps, axons injured by the CL reside in the proximal stump and nascent nerve bridge with macrophages throughout the 7 d conditioning period. Indeed, CD68^+^ CL macrophages can be seen in close proximity to SCG10 labeled regenerating axons in the proximal stump of transected *Ccr2*^*gfp*^ het and *Ccr2*^*gfp*^ KO nerves 4 and 7 d after injury (Fig. 5A-D). Thus, we examined Arg1^+^ CL site macrophages 9 d post injury in the transection CL paradigm (Fig. 5E) by staining for Arg1 and CD68 to identify lesion site macrophages, and for GFP to identify CCR2^+^-derived MDMs (Fig. 5F-S). In *Ccr2*^*gfp*^ hets, most CL site macrophages are MDMs (i.e., are GFP^+^) and express Arg1 (Fig. 5F-J, P-S); however, in *Ccr2*^*gfp*^ KOs, Arg1^+^ macrophages are still abundant but there are very few MDMs (Fig. 5K-O, P-T). While there is an overall reduction in CD68^+^ macrophages in the CL site of *Ccr2*^*gfp*^ KOs compared to hets (Fig. 5P), an increase in the number of GFP^−^CD68^+^ macrophages (Fig. 5Q) partially compensate for the loss of MDMs in the KO mice. The relative M2 macrophage number is also maintained (Fig. 5S) due to increased Arg1 expression among residents (Fig. 5T), suggesting they are adopting the appropriate phenotype. These observations are consistent with a model suggested by the TL site data (Fig. 4) that MDMs are preferentially recruited to lesion sites, but residents eventually migrate to and proliferate at a lesion site compensating for impaired recruitment.

**Fig. 6:**
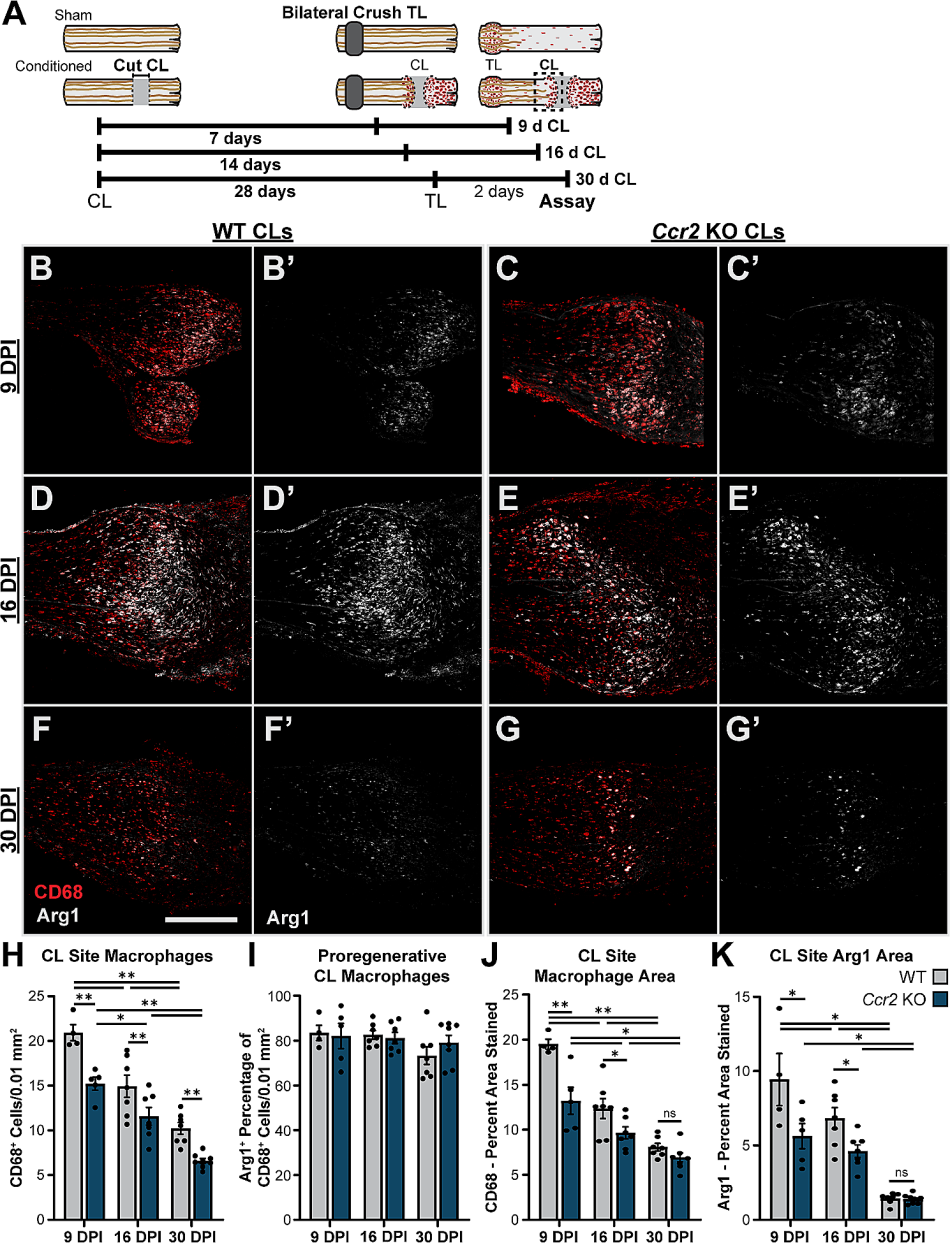
Arg1^+^ macrophages are still observed in the CL sites of both WT and *Ccr2* KO animals at 30 d after the CL. **A.** Diagram showing the lesion paradigms used with the dotted box indicating the location quantified in B-K. **B-G’.** Representative images of Arg1^+^ macrophages in WT and *Ccr2* KO CL sites from 7 d CL (B-C’), 14 d CL (D-E’), and 28 d CL (F-G’) paradigms displayed as CD68 and Arg1 colabeling (B-G) and Arg1 alone (B’-G’) in 10 μm sections. **H.** CL site macrophages were quantified by counting CD68 labeled cells within two 300 μm diameter circles centered on the inflammation at the nerve end and expressed as CD68^+^ cells per 0.01 mm^2^. **I.** Arg1^+^ macrophages were quantified by counting cells with immunofluorescence staining that colocalized with CD68^+^ cells and expressed as the percentage of Arg1^+^ CD68^+^ cells. **J.** Macrophages quantified by measuring the percent area of the CL labeled by CD68 which represents relative macrophage area. **I.** Total Arg1 expression quantified by the percent area of the CL labeled by Arg1. Scale bar = 500 μm. * *p* < 0.05. ** *p* < 0.01. *N* = 4–8 per group

To further evaluate the time course of compensation in CCR2 nulls and ascertain the longevity of Arg1^+^ CL macrophages, the CL site in WT and *Ccr2* KO mice 9, 16, and 30 d post-injury were stained for CD68 and Arg1 (Fig. 6A-G). At every time point, the number of CD68^+^ macrophages in *Ccr2* KO CLs was 75–80% of WT (Fig. 6H), although the number is maximal at 9 days and declines at a similar rate in both genotypes. This suggests that an equilibrium was reached in both genotypes rather than inefficient recruitment mechanisms causing a delayed inflammatory response in *Ccr2* KOs. Notably, the Arg1^+^ percentage of macrophages was equivalent between genotypes at all time points (Fig. 6I) despite being derived from different sources, which implies the lesion environment and not the macrophage lineage determines the phenotype. Macrophages in the *Ccr2* KO CLs also occupy less relative area than in WT CLs (Fig. 6J). However, the difference in macrophage area between genotypes diminishes over time (Fig. 6J) implying that the *Ccr2* KO macrophages are slightly larger on average which may be another compensatory mechanism. The CL site macrophages persist to some extent for at least 30 d (Fig. 6F-H) along with CL-enhanced regeneration (Fig. 1 and S2) in both WT and *Ccr2* KO animals. The macrophage response and Arg1 expression are lessening at the 30 d time point (Fig. 6F-K), raising the possibility that the CL response may also fade at a later time point, possibly due to resolution of the macrophage response.

### Activated macrophages in the sciatic nerve do not enhance regeneration

**Fig. 7:**
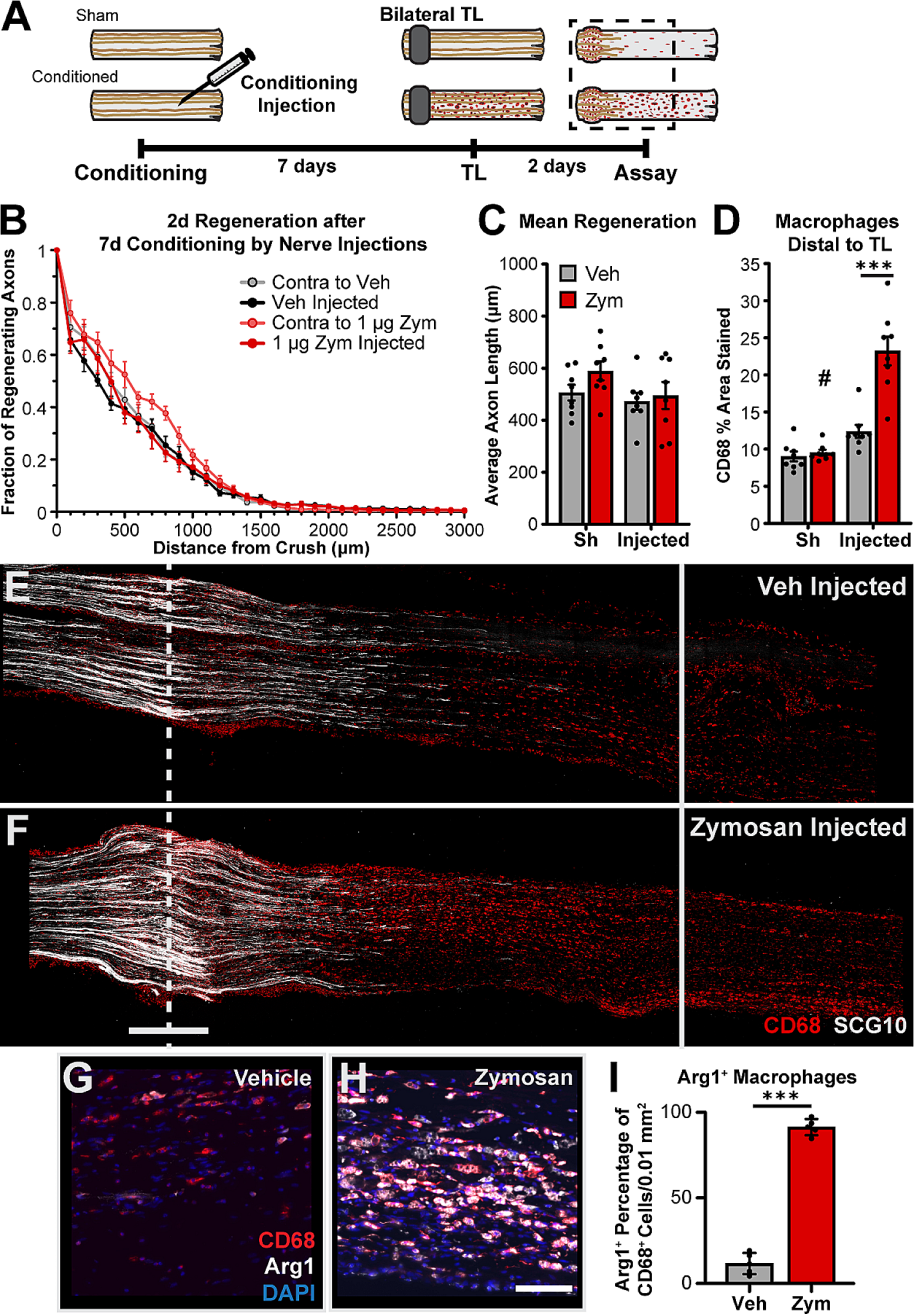
Zymosan induced recruitment of macrophages into the sciatic nerve does not condition axons. **A.** Diagram of the zymosan conditioning paradigm in which WT sciatic nerves received a unilateral conditioning injection of zymosan (Zym) or vehicle (Veh) 7 d before the nerves were given bilateral crush TLs and assayed after a 2 d regeneration period. **B.** Axon regeneration quantified at 100 μm intervals as the fraction of regenerating axons relative to the crush site using SCG10 staining. **C.** Mean regeneration distance calculated by integrating SCG10 immunofluorescent staining of regenerating axons. **D.** Macrophages in the 3 mm immediately distal to the TL measured by the percent area immunostained for CD68. **E-F.** Representative images of regenerating nerves immunostained for regenerating axons with SCG10 and macrophages with CD68 in 40 μm sections. The dotted line indicates the center of the crush site which was considered to be 500 μm wide, and the solid line is 3000 μm from the crush. Scale bar = 500 μm. Forty-micron sections were used in B-E. **G-H.** Vehicle and zymosan injected nerves were stained for CD68, Arg1, and DAPI. **I.** Arg1 expressing macrophages were quantified by the percentage of CD68^+^ cells that express Arg1 per 0.01 mm^2^. Scale bar = 100 μm. # indicates a significant (*p* < 0.05) difference between the Sh (unconditioned) and Injected (conditioned) regeneration within treatment groups (vehicle or zymosan). *** *p* < 0.001. *N* = 4–8 per group

Lesion site macrophages are a spatially unique population of immune cells responding to nerve injury, in that they are preferentially monocyte-derived, have a potentially pro-regenerative Arg1^+^ phenotype, and are present in every nerve that demonstrated enhanced regeneration; all characteristics of a population that could induce CL-enhanced regeneration. Therefore, we hypothesized that recruiting MDMs into an uninjured sciatic nerve could increase axon regeneration after a subsequent crush injury. To accomplish that we used zymosan, a yeast cell wall extract, which recruits and activates macrophages within nervous tissues [72], and increases the speed of functional recovery when injected into an injured sciatic nerve [41]. Additionally, zymosan-activated macrophages can enhance regeneration of sensory neurons in vitro and in vivo over long distances in the spinal cord [67, 72, 73]. To test if zymosan can induce a CL-like effect, again our CL paradigm was modified. Instead of a transection CL, the sciatic nerve was injected with either 1 µg zymosan (at 5 µg/µl) or vehicle, with a 7 d conditioning period before performing crush TLs with a 2 d regeneration period (Fig. 7A). The zymosan injection induced a robust inflammatory response throughout the segment distal to the TL (Fig. 7D-I) but did not enhance axonal regeneration (Fig. 7B, C, E, F). Interestingly, approximately 95% of zymosan-recruited macrophages, 1 mm distal to the crush site, express Arg1 (Fig. 7G-I). Since zymosan-activated macrophages can enhance regeneration in sensory neurons [67, 72, 73], these results indicate that inducing macrophage infiltration into the uninjured sciatic nerve is not sufficient to induce a CL-like regeneration enhancement in uninjured axons.

### CL-enhanced regeneration is resistant to alterations in CL macrophage phenotype

Lesion site macrophages express Arg1, a marker of anti-inflammatory M2 macrophages [74]. Arg1, along with inducible nitric oxide synthase (iNOS), are two of the most well documented macrophage polarization markers for anti-inflammatory/M2 and pro-inflammatory/M1 macrophages, respectively [44]. These markers are widely used because they are enzymes in competing pathways that utilize arginine as a substrate to produce either trophic polyamines via Arg1 or the inflammatory radical nitric oxide via iNOS [75]. The Arg1 inhibitor, nor-NOHA, can produce pro-inflammatory macrophage polarization in vivo [76]. Since M2 macrophages have been characterized as pro-regenerative for neurons [67], it was of interest to test if shifting the macrophages at the CL site to an inflammatory M1 phenotype would result in loss of regenerative support from macrophages and a reduction in CL-enhanced regeneration.

**Fig. 8:**
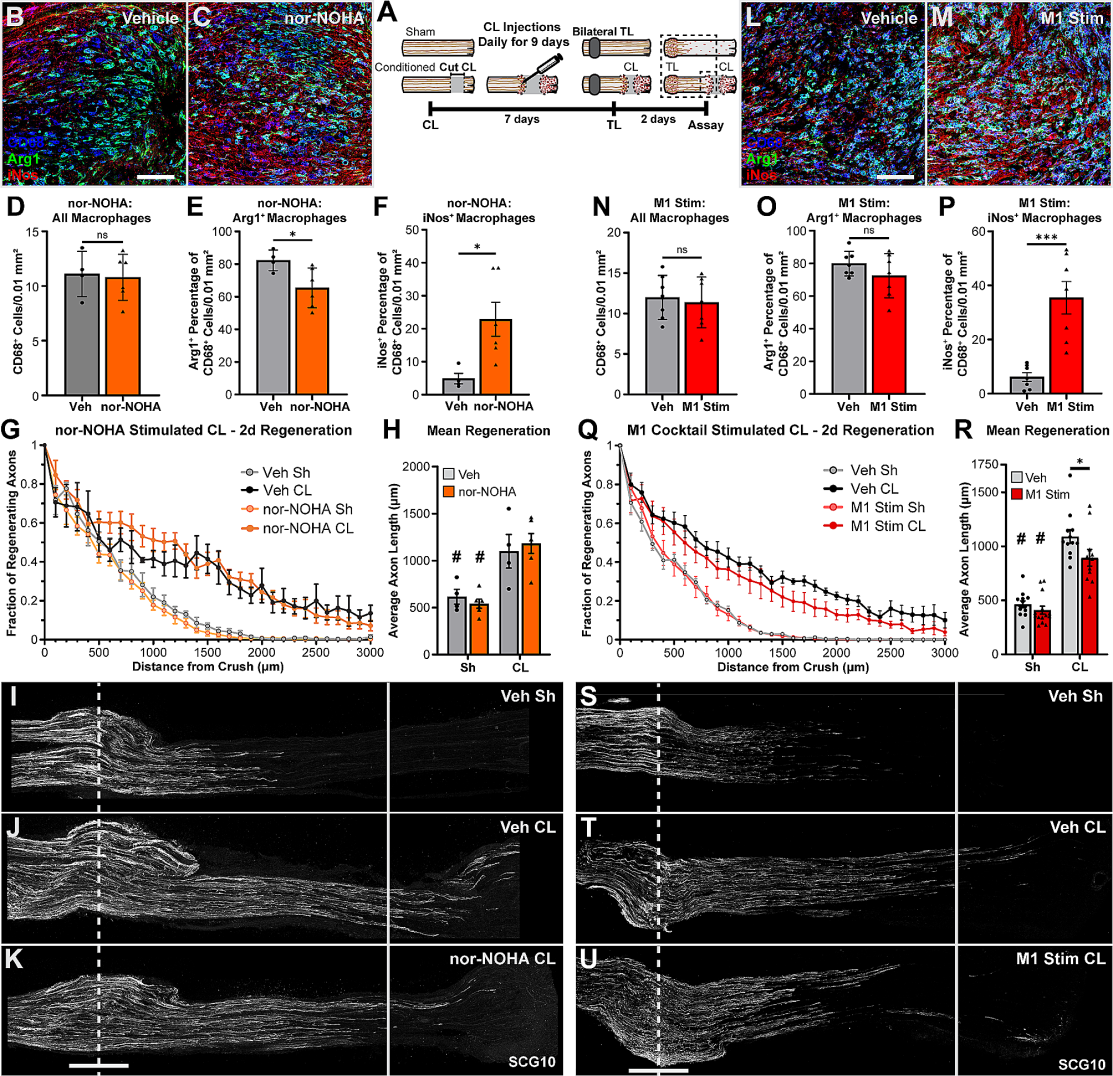
Effect of driving CL-macrophages toward an M1 phenotype on CL-enhanced regeneration. **A.** Diagram showing the CL paradigm performed on WT animals with daily CL injections to pharmacologically inhibit an M2 phenotype and promote an M1 phenotype at the CL. The arginase inhibitor, nor-NOHA (left, B-K), or an M1 stimulation cocktail, containing LPS and a STAT6 inhibitor (right, L-U), was injected daily into the CL site to polarize the macrophages from M2 to M1. **A-B, L-M.** Representative images for vehicle and arginase inhibitor (A-B), or M1 stimulation cocktail (L-M) treated mice showing CD68, Arg1, and iNOS, immunostaining. CD68 is shown in blue so that macrophages co-labeled with Arg1 will appear Cyan and those co-labeled with iNOS will appear magenta. Triple labeled cells will appear white. Note the increase in magenta cells and decrease in cyan cells in the nor-NOHA treated CL (C), compared to the increase in white cells with few magenta cells in the M1 stim CL (M). Scale bar = 100 μm in A, B, L, M. **D, N.** Macrophage number, displayed as cells per 0.01 mm^2^, was not altered by the treatment. **E, O.** The percentage of CD68^+^ macrophages expressing Arg1 after the arginase inhibitor (E) or M1 stimulation cocktail (O) treatment compared to vehicle controls. **F, P.** The percentage of CD68^+^ macrophages expressing iNOS after arginase inhibitor (F) or M1 stimulation cocktail (P) treatment compared to vehicle. **G, Q.** Axon regeneration quantified at 100 μm intervals as the fraction of regenerating axons relative to the crush site for arginase inhibitor (G) or M1 stimulation (Q) treated nerves. **H, R.** Mean regeneration distance calculated by integrating SCG10 immunofluorescent staining of regenerating axons for arginase inhibitor (H) or M1 stimulation (R) treated nerves. **I-K, S-U.** Representative images of regenerating nerves treated with arginase inhibitor (I-K) or M1 stimulation cocktail (S-U) immunostained for regenerating axons with SCG10 in 40 μm sections. Unconditioned regeneration (I, S) was the same for both treatment groups. Conditioned regeneration (J-K, T-U) was also the same between treatments and significantly increased compared to contralateral uninjected nerves. The dotted line indicates the center of the crush site which was considered to be 500 μm wide, and the solid line is 3000 μm from the crush. Scale bar = 500 μm in I-K, S-U. * *p* < 0.05. *** *p* < 0.001. # *p* < 0.05 between injury groups within the same treatment group. *N* = 4–11 per group

One of two treatments were injected daily into the CL site to alter the M2 phenotype (Fig. 8A). First nor-NOHA, an arginase inhibitor, was used. It did not alter the number of CD68^+^ macrophages at the CL site (Fig. 8B-C) but did result in a small decrease in the percentage of Arg1^+^ macrophages (Fig. 8E, B-C cyan or white cells) and a substantial increase in the percentage of iNOS-expressing macrophages (Fig. 8F, B-C magenta or white cells). However, treated mice displayed a CL response that was unaltered compared to vehicle treated controls (Fig. 8G-K). The second treatment was an “M1 stimulation cocktail” containing LPS, which is sufficient to induce the classical M1 phenotype in culture [44, 77], and an inhibitor of STAT6, which is a transcription factor that helps produce the M2 phenotype and inhibits the LPS response [47]. The M1 stimulation cocktail did not alter the number of CL macrophages or Arg1 expression but it did significantly increase CD68^+^ macrophage expression of iNOS (Fig. 8L-P, G-K magenta or white cells) perhaps to a slightly greater extent than did nor-NOHA. The preserved Arg1 expression with an increase in iNOS expression consequently manifests as an increase in triple-positive (white) cells. Interestingly, there was a small decrease in CL-enhanced regeneration in the M1 stimulated nerves compared to vehicle (Fig. 8Q-U); however, there was also still significantly enhanced regeneration compared to sham. These two treatments, which were both intended to inhibit an M2 and promote an M1 phenotype, altered the CL macrophage phenotypes in slightly different ways and to slightly different degrees but did not change Arg1^+^ macrophages into iNOS^+^ macrophages, demonstrating that macrophage phenotypes in vivo are more complex than an M1/M2 dichotomy. The modest inhibition of regeneration from the M1 stimulation suggests macrophages can impair regeneration, but it is unclear whether this is due to an impaired CL response or if the macrophages are creating an environment hostile to axons.

### Inducing apoptosis to deplete CL macrophages does not disrupt CL-enhanced regeneration

Since the CL macrophage phenotype does not seem to be necessary for CL-enhanced regeneration, we attempted to directly test their necessity by specifically ablating these macrophages with clodronate liposomes. Clodronate is a small molecule that acts intracellularly to induce apoptosis. By encapsulating it in liposomes, its toxic activity can be restricted to phagocytic cells, predominantly macrophages [49, 78], and clodronate liposomes have been used to deplete infiltrating macrophages in nerve injury models [79–81]. To restrict the activity of the clodronate further, clodronate or control liposomes were injected directly into the proximal CL site in a transection CL paradigm to target CL macrophages. The injections were given daily from the time of the CL until the animals were sacrificed at the end of our transection CL paradigm. Surprisingly, the CL sites seemed minimally affected and CD68^+^ cells were still abundant in the clodronate treated group. The cells appeared more numerous, but smaller, and rounder, likely explaining the slight decrease in CD68 area (Fig. S4 F-H) and suggesting that they were monocytes or neutrophils infiltrating in response to clodronate induced apoptosis of CL macrophages. CL-enhanced regeneration was also not inhibited in clodronate treated CLs (Fig. S4 A-E). However, since clodronate seemed to be causing localized CL macrophage apoptosis, we were able to use it to create another paradigm that nearly completely depleted CL macrophages.

We have shown that lesion site macrophages are predominantly MDMs but that residents can compensate relatively quickly and completely in the absence of CCR2-dependent recruitment (Figs. 4, 5 and 6). Importantly, MDM recruitment is also almost completely inhibited in *Ccr2*^*gfp*^ KOs [Figs. 5 and 35]. However, some CCR2-independent recruitment mechanisms may remain [35]. We also previously found that resident macrophages are a major chemokine source to recruit MDMs in the DN [35], and so they may also be the source of CCR2 independent recruitment signals. Thus, we hypothesized that removing residents and CCR2 signaling in the DN would completely abolish the macrophage response. To test this, a single dose of control or clodronate liposomes was injected into the 2 mm of nerve distal to a sciatic nerve transection in *Ccr2*^*gfp*^ Het and KO mice, and the DNs were examined 7 d after injury (Fig. S5 A-D). Clodronate liposome injected *Ccr2*^*gfp*^ het mice showed a small but significant decrease in macrophages distal to the injury site compared to vehicle controls (Fig. S5 E). However, ∼80% of macrophages in the nerve were GFP^+^, suggesting that macrophages were still being recruited to the nerve (Fig. S5 G). Strikingly, in clodronate liposome treated *Ccr2*^*gfp*^ KO mice, both GFP^+^ and GFP^−^ macrophages were still nearly completely absent 7 d after injury and significantly reduced compared to all other groups (Fig. S5 F-G), indicating that ablating residents prevents the DN macrophage response in *Ccr2* null animals. Most of the remaining macrophages in the clodronate treated *Ccr2*^*gfp*^ KO mice were GFP^−^ (Fig. S5 F), possibly residents migrating from beyond the injected area.

**Fig. 9:**
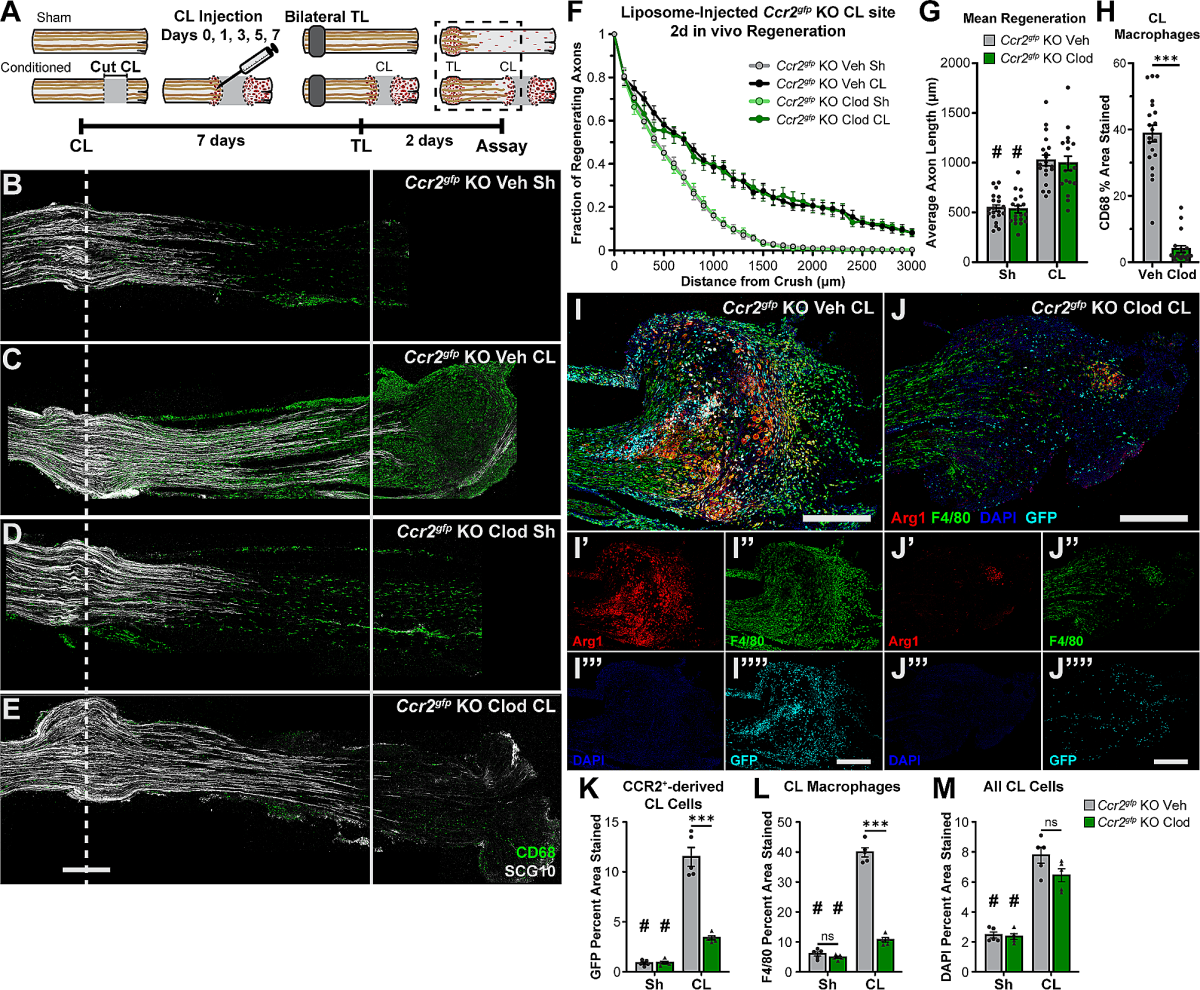
Clodronate liposomes injected into the CL site of *Ccr2*^*gfp*^ KO mice significantly reduces macrophages but does not prevent the peripheral CL response. **A.** Diagram depicting a CL paradigm performed on *Ccr2*^*gfp*^ KO animals in which CL macrophages were targeted for ablation by injecting clodronate liposomes into the proximal CL site on the day of the CL (day 0) the next day (day 1) and every other day thereafter. Control animals received vehicle liposome injections instead. **B-E.** Representative images of regenerating nerves immunostained for regenerating axons with SCG10. Unconditioned regeneration (B, D) was the same for both treatment groups. Conditioned regeneration (C, E) was also the same between treatments and significantly increased compared to contralateral uninjected nerves. The dotted line indicates the center of the crush site which was considered to be 500 µm wide, and the solid line is 3000 µm from the crush. Scale bar is 500 µm. **F.** Axon regeneration quantified at 100 µm intervals as the fraction of regenerating axons relative to the crush site. **G.** Mean regeneration distance calculated by integrating SCG10 immunofluorescent staining of regenerating axons. **H.** Macrophages quantified by percent CD68 positive area in a circle placed on the CL with a diameter equal to the largest width of the uninjured portion of the nerve. Forty-micron sections were used in B-H. **I-J.** Representative images of a PBS (vehicle) liposome-treated (I) and clodronate liposome-treated (J) *Ccr2*^*gfp*^ KO CL site immunostained for macrophages with F4/80, proregenerative macrophages with Arg1, recruited CCR2^+^ macrophages with GFP, and DAPI. F4/80 is a membrane marker, shown in green, while Arg1, shown in red, and GFP, shown in cyan, are both intracellular. These colors cause proregenerative F4/80^+^, Arg1^+^ macrophages to appear yellow or red ringed by yellow, recruited proregenerative GFP^+^, Arg1^+^ monocyte/macrophages to appear cyan and white, and triple positive cells to appear white surrounded by yellow-green. **I’-J’’’’.** Individual channel images from I and J. Percent area stained was quantified by outlining the tissue of the entire CL site. **K.** GFP staining was significantly reduced within the CL site of clodronate liposome treated *Ccr2*^*gfp*^ KO mice compared to vehicle treated mice. **L.** F4/80 macrophage staining was also significantly reduced in clodronate liposome versus PBS liposome treated mice. **M.** Despite the significant reduction in macrophages in the CL site of clodronate liposome treated mice, DAPI staining was not altered in the CL site. Scale bar is 500 μm. *N* = 18–20 per group for B-H and *N* = 5 per group for I-M. ** *p* < 0.01; *** *p* < 0.001. # *p* < 0.05 between injury conditions within the same treatment group

These data support a model in which macrophages responding to a nerve injury are recruited by the fastest available mechanism. Under normal circumstances this is recruiting MDMs via CCR2 which can still maintain an inflammatory response even when macrophages are being constantly depleted (Fig. S4). When the rapid CCR2 dependent mechanism is lost, resident macrophages and alternate recruitment mechanisms are able to compensate eventually. Additionally, lesion site macrophage accumulation seems to have more robust compensatory mechanisms than the DN (Figs. 4, 5 and 6, S5). Thus, to deplete CL macrophages, residents need to be removed by injecting clodronate, and MDM recruitment needs to be prevented by using *Ccr2* null animals. To test this, *Ccr2*^*gfp*^ KO mice underwent a transection CL paradigm with injection of either clodronate or vehicle liposomes into the proximal CL on days 0 (with the CL), 1, 3, 5, and 7 (with the TLs) before sacrificing on day 9 (Fig. 9A). As hypothesized, macrophages in the CL site were at least 80–90% depleted after clodronate treatment as measured by GFP, F4/80, and CD68 staining (Fig. 9H-L; Fig. S6 A, C, E). F4/80 staining is poorly colocalized with GFP staining (Fig. 9J) and CD68 staining in the adjacent section (Fig. S6 C, arrowheads) raising the novel possibility that there is a population of F4/80 expressing cells in the nerve that are not from a myeloid lineage. Despite the substantial macrophage depletion, there was minimal change in the total cell number measured by DAPI staining (Fig. 9M). Indeed, there was an increase in Ly6G staining at the CL site, indicating an increase in neutrophils (Fig. S6 B, D, F) which may be compensating for the decrease in macrophages and which colocalized with some of the remaining CD68 signal (Fig. S6 C, D arrowheads). Since these are *Ccr2*^*gfp*^ KO animals, TL macrophages are also nearly completely depleted (Fig. 4 and illustrated in Fig. 9C, dotted line). Surprisingly, however, CL-enhanced regeneration was not impaired in the clodronate injected nerves (Fig. 9B-G) demonstrating that lesion site macrophages are not necessary for CL-enhanced regeneration. Further, this was again our transection CL paradigm which prevents reattachment of the proximal and distal stumps. Additionally, in this paradigm, the proximal CL was visualized and mobilized every other day for injection ensuring that the severed axons in the proximal stump and the degenerating DN were always separated by several millimeters. Thus, there was no opportunity for the DN to promote regeneration through providing a supportive substrate or through juxtacrine or paracrine signaling.

## Discussion

Signals derived from the injured nerve and the macrophages found there [17, 21] have been proposed to enhance regeneration and the CL effect. This is the first study to investigate the various macrophage subtypes found in the injured nerve and their effect on in vivo regeneration. Using *Ccr2* null models, we interrogated the role of MDMs and found that the WD-independent inflammation at a lesion site relies on CCR2 signaling but that resident macrophages and CCR2-independent MDM recruitment are rapidly able to compensate. Further, we show the lesion site macrophages are a long-lasting population with a unique M2-like phenotype that is independent of their lineage. To test their effect on regeneration, WD-dependent and -independent macrophages in the nerve were systematically depleted by combining *Ccr2* null genetic models with surgical and pharmacologic techniques. MDMs, and TL macrophages in particular, were depleted with *Ccr2* null animals, the DN environment with all WD-dependent inflammation was physically prevented from interacting with injured axons using transection CLs, and WD-independent macrophages at the CL were depleted using clodronate injections into the CL of *Ccr2*^*gfp*^ KO animals. There was no difference in CL-enhanced regeneration between *Ccr2* KO transection CL nerves and WT crush CL nerves showing that the CL response does not require MDMs or the interaction of axons with the WD environment in the DN. Further, even when injured axons do not interact with any populations of injury-induced nerve macrophages, both unconditioned and CL-enhanced regeneration are unimpaired indicating these macrophages are not necessary for the CL effect or axon regeneration.

### *Ccr2* KOs and removal of Wallerian degeneration-dependent inflammation does not impair regeneration enhancement

Previous publications utilizing the Wallerian degeneration slow (Wld^s^) mouse or the *Sarm1* KO mouse, which both display significantly delayed WD, still show a robust immune response including neutrophils, MDMs, and resident macrophages at the site of injury [63, 64]. In the *Sarm1* KO mouse, two waves of inflammation involving macrophages have been demonstrated in the injured sciatic nerve [64]. The first is a response at the site of nerve injury, which is maximal by 3 to 5 d post injury, restricted to the injury site, and independent of WD. The second wave is maximal at 7 to 9 d post injury and is localized to the entirety of the DN [64].

MDMs recruited by CCR2 are the major component of WD-dependent inflammation [35, 82] and were thought to be necessary to enhance regeneration [33]. To determine if WD-dependent inflammation played a direct or indirect role in the CL response, a crush CL was used to leave the epineurium and other ECM components of the nerve intact. Axons grow into the nerve distal to the crush by 2 d post-injury, evidenced by the sham nerve growth in all our experiments, and thus interact with the DN environment during the conditioning period. Neither reducing DN macrophages, by performing a crush CL in *Ccr2* KOs, nor completely removing the DN environment, by performing transection CLs in WT and *Ccr2* KOs, decreased CL-enhanced regeneration compared to the WT crush CL. Finally, we showed that myelin removal, also thought to promote enhanced regeneration, was not necessary for regeneration enhancement, although it likely still has a small benefit for conditioned axons [7]. Further, the minimal myelin removal in the regenerating segment during the 2 d regeneration period suggests WD had not yet begun. However, any changes that did occur were not sufficient to enhance regeneration, evidenced by the comparatively slow regeneration in the unconditioned sham nerves. If WD-dependent inflammation served as a necessary source for retrograde injury signals required for regeneration [83], or influenced the regenerative response through alternative mechanisms, a change in the CL response elicited by a crush CL versus a cut CL would be apparent.

MDMs and the DN environment also were not necessary for maintenance of CL-enhanced regeneration in the peripheral or central branch. We extended the conditioning period from the normal 7 d to 28 d to test if WD-dependent inflammation and MDMs were needed to maintain the CL response. A transection CL maintained CL-enhanced regeneration in the sciatic nerve for 4 weeks indicating that neither the DN environment nor MDMs and CCR2 signaling are needed to enhance regeneration peripherally. We also tested the necessity of these macrophage subsets in supporting CL-enhanced regeneration of injured dorsal root axons. The possibility existed that different signaling and/or non-neuronal support were necessary for CL-enhanced regeneration of normally regeneration-deficient dorsal root. Again, WT and *Ccr2* KO mice displayed equivalent levels of CL-induced regeneration of the dorsal root 3 d post-crush injury.

The *Ccr2* KO mouse, which has an approximately 50% reduction of macrophages in the sciatic nerve after injury [18, 19, 82] displayed no difference in axonal regeneration compared to WT after either a 2 d crush injury or a CL. It was surprising that CCR2 signaling was not needed for enhanced regeneration as such a dependence was previously reported in culture experiments [18, 33], and it is unclear what important differences exist between the in vitro and in vivo assays, as both were performed in the same way up until the TLs. The quantification methods for axonal growth may contribute to the discrepancy. For in vivo regeneration, we have derived a method to quantify the theoretical average axon length without measuring individual axons [35]. In vitro, DRG explants were quantified by counting the 20 longest neurites, and dissociated cultures were quantified by measuring the longest axons of neurons that had grown a certain minimum distance [18]. These latter two methods quantify a subset of neurons with the most robust growth and thus will tend to represent the growth of an outlier population. That population may be representative of a true biologic subset of neurons that respond to cues induced by CCR2 signaling but which do not represent the whole population of conditioned neurons.

To examine the effect of macrophages on the initial period of peripheral nerve regeneration and the importance of the local environment for the CL response, sciatic nerves were conditioned with zymosan 7 d prior to a crush lesion. Zymosan, when injected into the DRG, has been shown to increase axonal regeneration of the central branch of unconditioned sensory neurons across the dorsal root entry zone and into the spinal cord [72]. Additionally, injection of zymosan into the intraocular space of the eye promotes significant regeneration of the injured optic nerve [84]. Despite achieving substantial recruitment of Arg1^+^ zymosan-activated macrophages in the nerve, there was no regeneration enhancement after a sciatic nerve crush injury compared to vehicle-treated controls, demonstrating that these macrophages can neither interact with uninjured axons to stimulate a CL-like effect nor alter the nerve environment to improve basal regeneration. Endogenous Arg1^+^ TL macrophages are also incapable of increasing regeneration of newly injured axons demonstrated by comparing WT and *Ccr2* null sham nerves. Throughout the CL paradigm, sham WT nerves are only ever exposed to TL macrophages while sham *Ccr2* null nerves never encounter macrophages, and yet the axons show equivalent growth. Thus, any benefits regenerating axons derive from nerve macrophages [21, 41] may come at later time points, possibly requiring induction of the CL response, or from other macrophage populations.

### Wallerian degeneration-independent inflammation

The peripheral transection CL paradigm utilized for most of this paper primarily tests the role of WD-independent inflammation. The nerve segment where regeneration is measured is proximal to the CL site and distal to the 2 d old crush TL. WD-dependent inflammation begins to occur 72 h [35] post injury and is maximal at 7 d [19, 32, 85], and thus is not present in the regenerating segment. Indeed, we confirm no myelin degeneration has begun in the regenerating segment we examine. Whereas, we show WD-independent inflammation at the lesion sites occurs more rapidly. Thus, macrophage accumulation and infiltration is mainly localized to the injury sites of WT mice in this nerve segment, while CCR2 null animals have absent TL macrophages and reduced CL macrophages.

We confirm here that the lesion site is a unique environment, which shapes the macrophages found there differently from those located in the DN compartment [17, 64]. Most macrophages in the CL site of both WT and *Ccr2* null animals express Arg1 which is both an M2/anti-inflammatory macrophage marker [86] and enzymatically promotes healing by metabolizing the nitrogen in arginine to produce polyamines and shunting nitrogen away from nitric oxide production [87]. The proportion of Arg1^+^ CL site macrophages is similar in WT and *Ccr2* nulls despite a significant reduction in MDMs in the *Ccr2* nulls, suggesting they share a phenotype and possibly a function despite being derived from different populations. This is a novel finding as macrophages derived from different sources often respond distinctly to the same set of signals [88] and suggests that the signals present in the lesion sites are potent drivers of a particular macrophage phenotype. Interestingly, lesion site macrophages have been shown to express high levels of efferocytotic receptors suggesting that clearance of dying cells may be the primary driver of their unique expression profile [17, 64].

### Sciatic nerve macrophages are not required for CL-induced peripheral axon regeneration

Clodronate liposomes are a well-used method to systemically and locally deplete monocytes and macrophages [89]. In WT mice, daily administration of clodronate liposomes to the CL only reduced CD68 staining at the CL site by ∼20% compared to PBS-liposome treated mice, indicating recruitment mechanisms are sufficient to overcome apoptosis. Further characterization of clodronate liposome treatment was carried out by performing a single injection of clodronate liposomes into the sciatic nerve 2 mm distal to a transection injury in *Ccr2*^*gfp*^ het and KO mice. *Ccr2*^*gfp*^ het mice treated with clodronate liposomes displayed a 41% reduction in CD68^+^ staining 7 d post-injury compared to PBS-liposome treated mice. Conversely, following a single injection, *Ccr2*^*gfp*^ KO mice treated with clodronate liposomes displayed a 79% reduction in CD68 staining compared to PBS liposome treated *Ccr2*^*gfp*^ KO mice. In CCR2-competent mice, it is likely that the continual apoptosis caused by the local clodronate treatment indirectly leads to recruitment of MDMs to the site of injury. Indeed, this is supported by the fact that 78% of macrophages in the clodronate treated *Ccr2*^*gfp*^ heterozygous mice express GFP, defining them as MDMs. In the absence of CCR2, this major recruitment mechanism is unavailable, explaining the 79% depletion at 7 days in *Ccr2*^*gfp*^ KO mice treated with clodronate liposomes compared to PBS liposomes. Thus, local clodronate administration can effectively eliminate macrophages in the *Ccr2* null sciatic nerve.

Given that MDMs are the primary source of macrophages at lesion sites in WT mice and that macrophages in the nerve are a major source of CCL2, the primary CCR2 ligand [35], we hypothesized that clodronate administration into the CL site of *Ccr2* null mice would yield a significant reduction in macrophages. When clodronate liposomes were injected into the CL site of *Ccr2*^*gfp*^ KO mice, lesion site macrophages were reduced by 80–90% in the CL site compared to PBS liposome controls as measured by either CD68 or F4/80 staining. Macrophages were also almost completely absent at the TL site in *Ccr2*^*gfp*^ KO mice, as discussed earlier, and since a transection CL was used, injured axons were prevented from interacting with the DN. Surprisingly, the CL effect in clodronate treated *Ccr2*^*gfp*^ KO mice was indistinguishable from the PBS liposome-treated mice and was essentially equivalent to CL-enhanced regeneration observed in all other experiments indicating that the DN environment and lesion site macrophages are not necessary for the CL response. This also implies that any nerve-derived signals required for the CL response must come from the proximal CL or proximal nerve and the cells found there.

The depletion of nerve macrophages was nearly complete in this CL paradigm with the only remaining macrophages consisting of at most 10–20% of CL macrophages. Our primary macrophage marker, CD68, can label other myeloid cells such as neutrophils and monocytes [69–71]. Indeed, depletion of CL macrophages led to a large increase in neutrophils measured by Ly6G staining which accounts for some of the remaining CD68 staining. Additionally, our staining revealed many F4/80^+^ cells which did not express GFP or CD68, indicating they were not derived from CCR2^+^ monocytes and likely not any myeloid lineage. They are primarily located in the nerve segment adjacent to the CL, and we have observed similar F4/80 profiles in the DN 2 and 3 d post-injury [35]. Their elongated morphology and presence in the nerve both proximal and distal to a lesion suggests that they are Schwann cells. Thus, while it is possible that the fraction of remaining nerve macrophages are inducing CL-enhanced regeneration, it is far more likely that another cell population, possibly Schwann cells, neutrophils, or macrophages in another compartment, are either compensating for the loss of nerve macrophages or are responsible for CL-enhanced regeneration.

#### Alternative sources of stimuli for CL-enhanced regeneration

Barrette et al. [21] demonstrated that depletion of myeloid cells in the lesioned sciatic nerve dramatically impairs regeneration in response to a single injury. While this paper is sometimes referred to as showing the importance of the nerve macrophage response to injury, the authors emphasize that they have depleted both macrophages and granulocytes. It is noteworthy, however, that neutrophil depletion by itself did not affect nerve regeneration from a single lesion [90]. The latter result together with our findings that depletion of nerve macrophages does not inhibit the CL response raises an important question about the interplay between neutrophils and macrophages within the injured nerve environment. Notably, a population of novel pro-regenerative neutrophils has been identified, which unlike other neutrophils are not depleted by antibodies to Ly6G and can induce a CL response in the dorsal columns after being injected into the sciatic nerve [91]. Thus, the compensatory increase in neutrophils we observe when CL macrophages are depleted could be involved in initiating or sustaining the CL response.

In the absence of resident and MDMs in the nerve parenchyma, other populations of macrophages could be compensating for their loss and support the CL response. Significant macrophage accumulation is observed in L (lumbar) 3–5 DRGs following a sciatic nerve injury. Proliferating resident macrophages and a small influx of MDMs lead to a 2-fold increase in macrophages by 3 to 7 d post-injury [16, 17, 34, 35]. A recent paper used a CSF1R inhibitor (PLX73086) to deplete macrophages in the DRG after injury, while maintaining a normal macrophage response in the sciatic nerve, and found that axonal regeneration after a single nerve crush injury was significantly reduced [16]. Additionally, single cell RNA-sequencing has revealed that epineurial macrophages are a distinct *Relmα*^+^*Mgl1*^+^ population of macrophages in the sciatic nerve that respond differently to injury than macrophages located within the nerve parenchyma [36]. Another defect revealed by Barrette et al. [21] was that angiogenesis in response to nerve injury, much of which occurs in the epineurium, was absent when myeloid cells were depleted. Macrophages within the DRG and/or epineurial macrophages in the sciatic nerve could play a significant role in the CL response through direct or indirect mechanisms.

### Role of M1 macrophages and the nerve environment

We have shown that induction of CL-enhanced regeneration occurs with or without the M2-like macrophages found in the CL and further cannot be induced by the recruitment of M2-like macrophages using Zymosan. This implies that M2 signals (and macrophage derived signals in general) in the nerve are not enhancing regeneration. However, we were able to impair CL-enhanced regeneration with our M1 stimulation cocktail containing a STAT6 inhibitor and LPS. This cocktail increased pro-inflammatory marker expression in CL macrophages without affecting the expression of M2 markers. Thus, this CL contained its own unique signaling environment with macrophage-derived M1 and M2 signals as well as the injected LPS and STAT6 inhibitor. This could have led to the modestly reduced regeneration through incomplete induction of the CL response, inhibition of the CL response, or creation of substances capable of inhibiting CL-enhanced axon growth. Since CL-enhanced regeneration is maximally induced in the absence of macrophages, it is likely that the impaired CL-enhanced regeneration is either due to the inhibitory nature of the environment or inhibitory signals. This could be further elucidated in the future by examining regeneration in vitro to remove any inhibition due to the environment, and by sorting and sequencing M1 stimulated macrophages to characterize their phenotype in detail.

## Conclusions

Here we provide clear evidence that both DN nerve and lesion site macrophages, defining features of WD-dependent and -independent inflammation, respectively, are not necessary for CL-enhanced regeneration in the sciatic nerve. These data have many implications for the future study of the CL response, such as the source(s) of specific retrograde injury signals including growth factors and gp130 cytokines, the functional significance of Arg1 expressing macrophages at the nerve lesion site, and the role of macrophages in other nervous system compartments in axonal regeneration.

### Electronic supplementary material

Below is the link to the electronic supplementary material.

Supplementary Material 1

## Data Availability

No datasets were generated or analysed during the current study.

## Declarations

The authors have no declarations.

(Approval Number: 2015-0009) and were conducted in accordance with the guidelines of the National Institutes of Health on the care and use of animals.

## Abbreviations

Arg1: arginase 1
CCR2: C-C motif chemokine receptor 2
CL: conditioning lesion
CNS: central nervous system
DAPI: 4′,6-diamidino-2-phenylindole
DRGs: dorsal root ganglia
iNOS: inducible nitric oxide synthase
KO: knock out
DN: distal nerve
KI: knock in
DPI: days post-injury
GFP: green fluorescent protein
LFB: luxol fast blue
LPS: lipopolysaccharide
MDM: monocyte-derived macrophage
nor-NOHA: alpha-amino acid N(omega)-hydroxy-nor-l-arginine
PNS: peripheral nervous system
PBS: phosphate buffered saline
RAG: regeneration associated gene
ROI: region of interest
SARM1: sterile alpha and TIR motif containing 1
SCG10: superior cervical ganglion 10 or stathmin 2
STAT6: signal transducer and activator of transcription 6
TL: test lesion
WD: Wallerian degeneration
WT: wild type
