# Supplementary material for: Neither injury induced macrophages within the nerve, nor the environment created by Wallerian degeneration is necessary for enhanced in vivo axon regeneration after peripheral nerve injury
Source: J Neuroinflammation. 2024 May 27;21:134. doi: 10.1186/s12974-024-03132-5 (PMC11131297; doi:10.1186/s12974-024-03132-5)
Supplement: Supplementary file 1 — Supplementary Material 1 [file 12974_2024_3132_MOESM1_ESM.docx]

**Supplementary Figures**


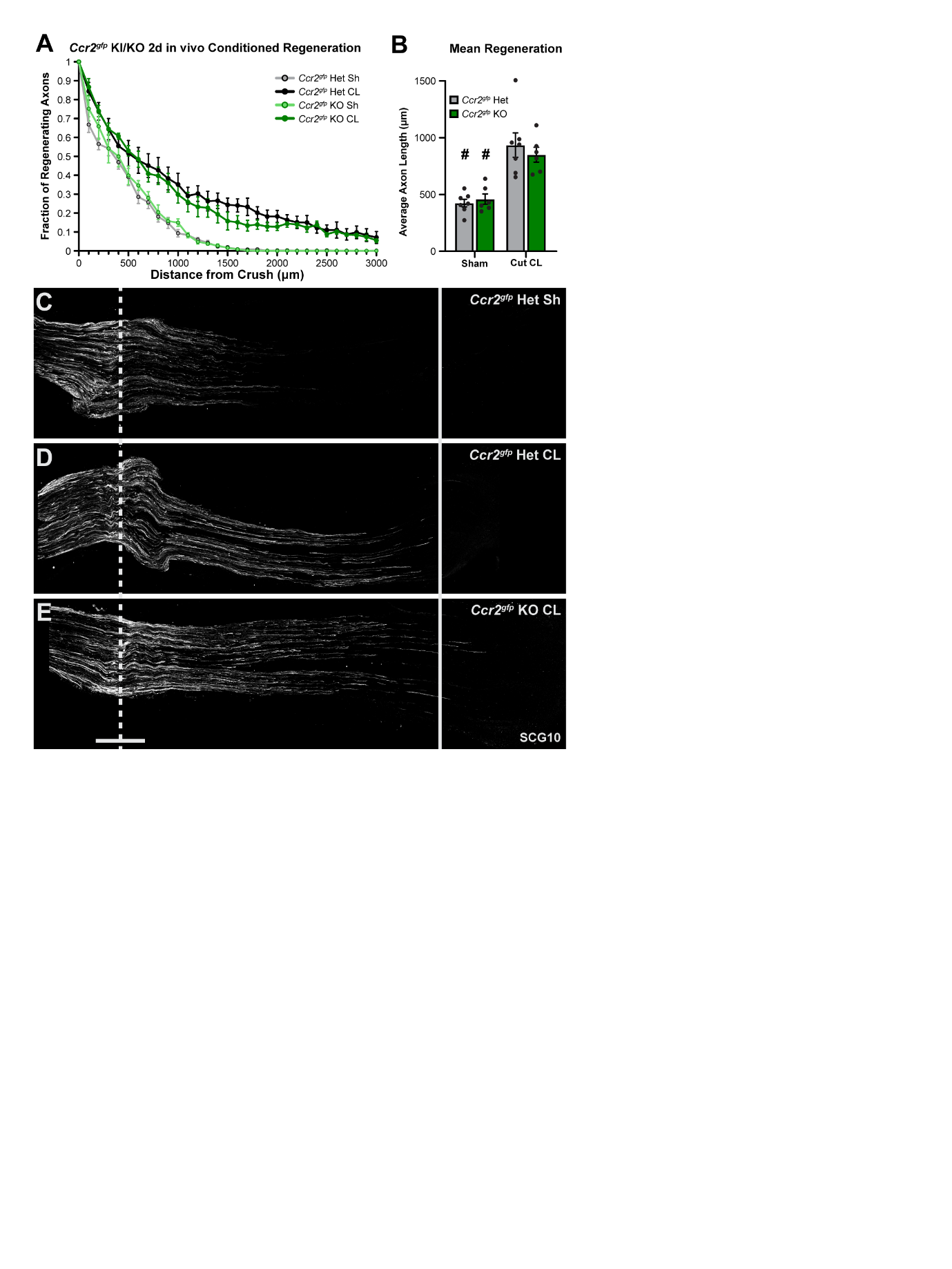


**Figure S1**

*Ccr2* KO animals still display normal CL-enhanced regeneration after a crush CL. *Ccr2^gfp^* animals underwent an in vivo Cut CL assay as in Fig. 1H **A.** Axon regeneration quantified at 100 µm intervals as the fraction of regenerating axons relative to the crush site for *Ccr2^gfp^* het and KO mice. **B.** Mean regeneration distance calculated by integrating SCG10 immunofluorescent staining of regenerating axons in *Ccr2^gfp^* het and KO mice. **C-E.** Representative images of regenerating nerves from *Ccr2^gfp^* het and KO mice immunostained for regenerating axons with SCG10 in 40 µm sections. The dotted line indicates the center of the crush site which was considered to be 500 μm wide, and the solid line is 3000 μm from the crush. Scale bar = 500 μm. # indicates a significant (p < 0.05) difference between the Sh (unconditioned) and CL (conditioned) regeneration within a genotype. N = 6–7 per group.


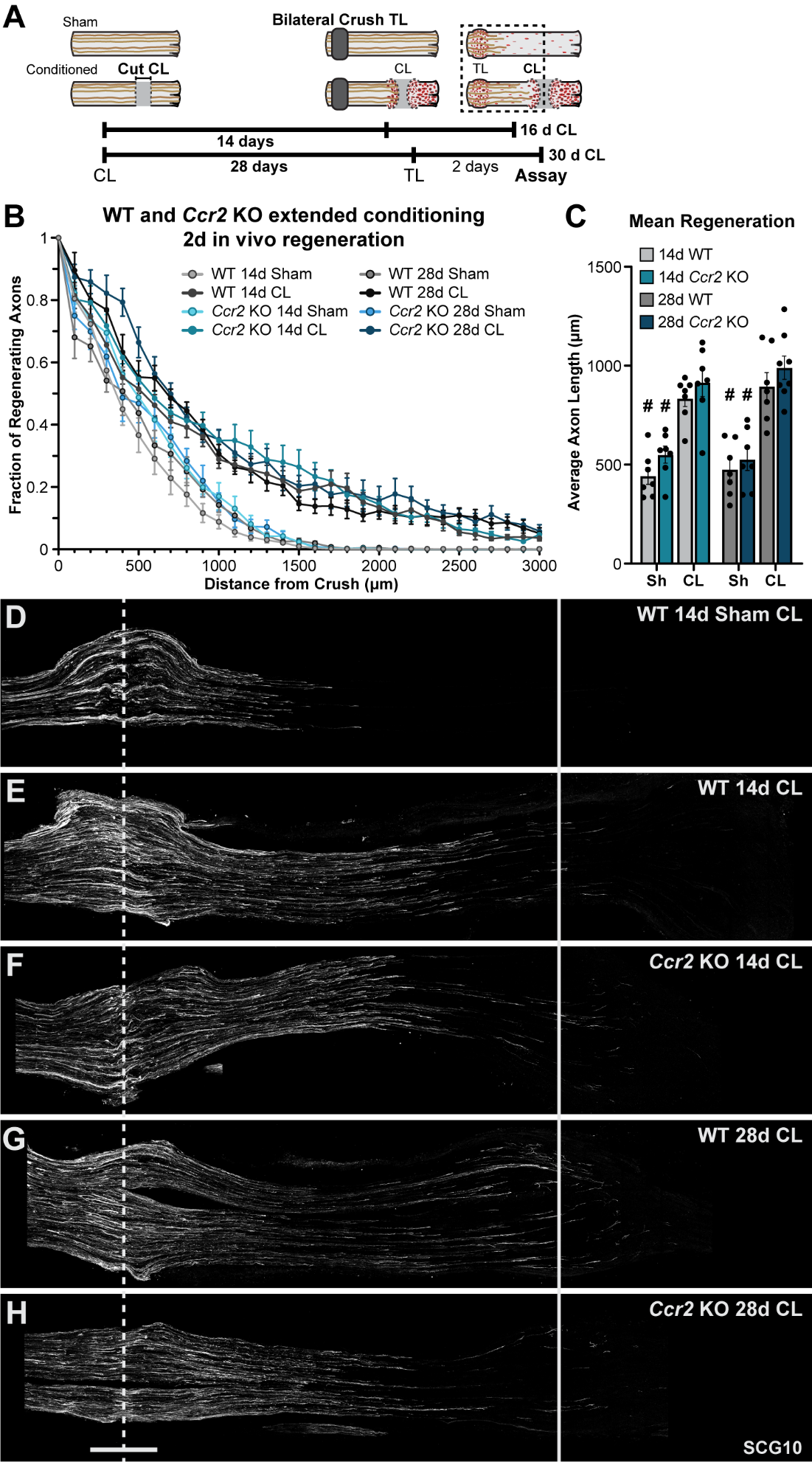


**Figure S2**

CL enhanced peripheral regeneration is maintained for at least 28 days after a CL. **A**. Diagram indicating WT and *Ccr2* KO animals underwent a Cut CL as in Fig. 1 except that the conditioning period was either 14 or 28 d. **B.** Axon regeneration quantified at 100 µm intervals as the fraction of regenerating axons relative to the crush site. **C.** Mean regeneration distance calculated by integrating SCG10 immunofluorescent staining of regenerating axons. **D-H.** Representative images of regenerating nerves immunostained for regenerating axons with SCG10 in 40 µm sections. Unconditioned regeneration (D) was the same for all groups. Conditioned regeneration (E-H) was also the same between genotypes and significantly increased compared to contralateral unconditioned nerves. The dotted line indicates the center of the crush site which was considered to be 500 μm wide, and the solid line is 3000 μm from the crush. Scale bar = 500 μm. # indicates a significant (p < 0.05) difference between the Sh (unconditioned) and CL (conditioned) regeneration within a genotype. N = 7 per group.


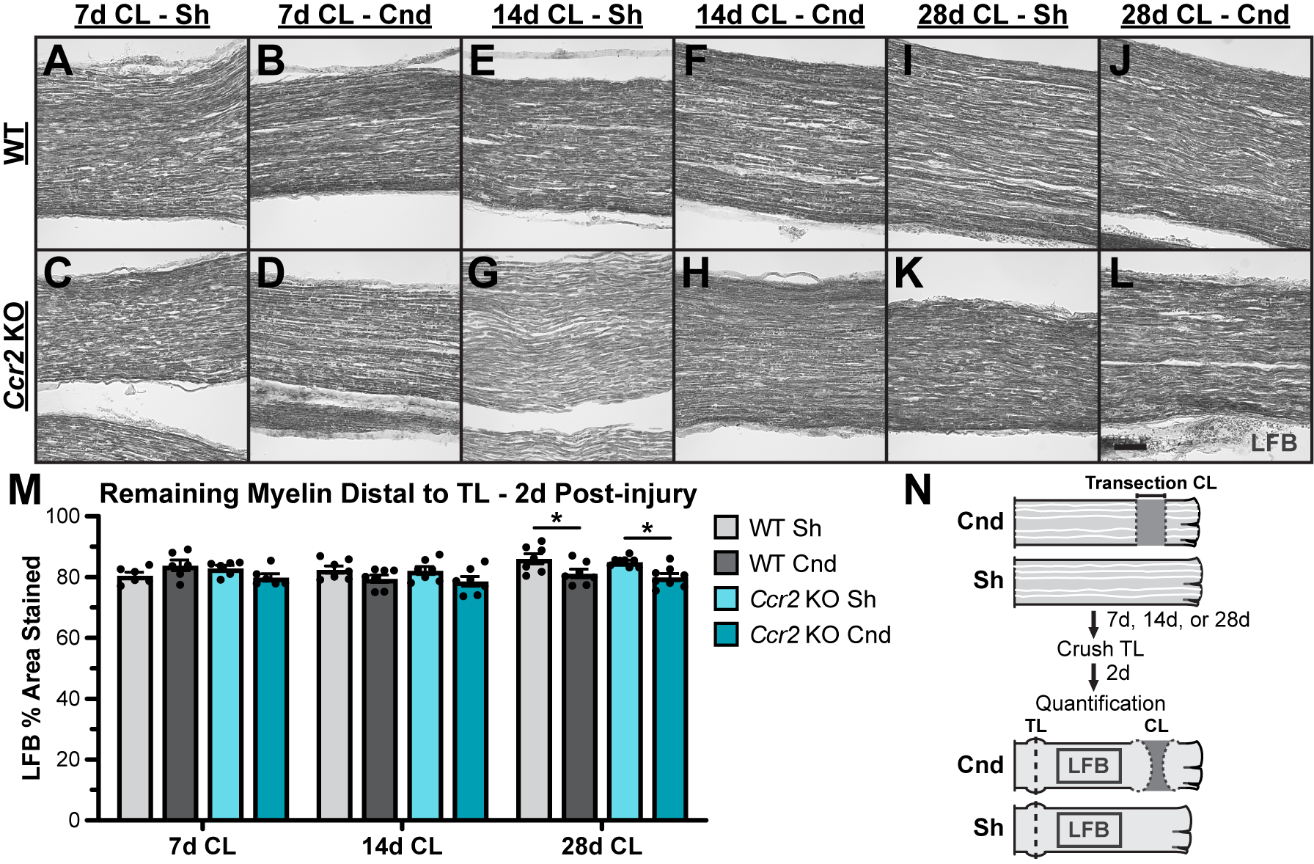


**Figure S3**

Myelin is not removed from the regenerating segment distal to the TL in either conditioned or unconditioned nerves of WT or *Ccr2* KOs during the 2 d regeneration period. **A-H.** Representative images of myelin distal to the TLs stained with LFB. Myelin clearance is unchanged in both genotypes or in the injury conditions of the 7 d (A-D) and 14 d (E-H) CL experiments. **M.** Quantification of myelin clearance by LFB area stained within the nerve shows a reduction in LFB staining at the 28 d time point between sham and conditioned nerves in both genotypes. **N.** Diagram of the injuries and area quantified. Cnd = conditioned. Scale bar = 100 μm. * indicates a significant difference (p < 0.05) between the indicated groups. N = 6–8 per group.


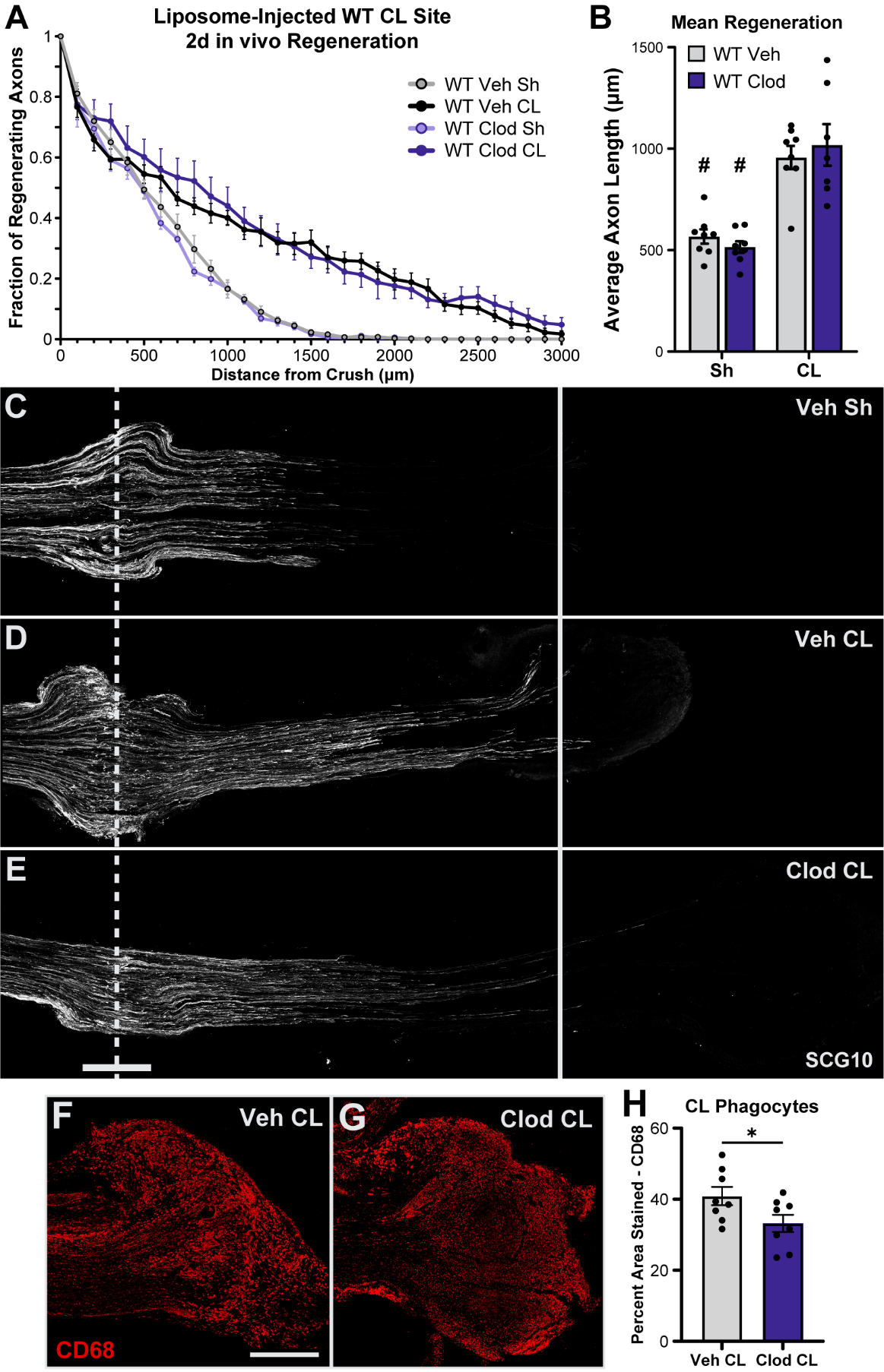
**Figure S4**

Clodronate liposomes injected into the CL site of WT mice disrupts macrophages but does not prevent monocyte recruitment. WT animals underwent an in vivo CL paradigm in which CL macrophages were targeted for ablation by injecting clodronate liposomes into the CL site daily. Control animals received vehicle liposome injections instead. **A.** Axon regeneration quantified at 100 µm intervals as the fraction of regenerating axons relative to the crush site. **B.** Mean regeneration distance calculated by integrating SCG10 immunofluorescent staining of regenerating axons. **C-E.** Representative images of regenerating nerves treated with either control or clodronate liposome injections, immunostained for regenerating axons with SCG10 in 40 µm sections. Unconditioned regeneration (e.g. C) was the same for both treatment groups. Conditioned regeneration (D-E) was also the same between treatments and significantly increased compared to contralateral uninjected nerves. The dotted line indicates the center of the crush site which was considered to be 500 μm wide, and the solid line is 3000 μm from the crush. Scale bar is 500 μm. **F-G.** Representative images of PBS and clodronate injected nerves stained for CD68. **H.** Macrophages quantified by percent CD68 positive area in a circle placed on the CL with a dimeter equal to the largest width of the uninjured portion of the nerve. Clodronate liposomes resulted in a small but significant decrease in CD68^+^ macrophage staining at the CL site. N = 8 per group. Scale bar = 500 μm. * *p* < 0.05. # *p* < 0.05 between injury conditions within the same treatment group.


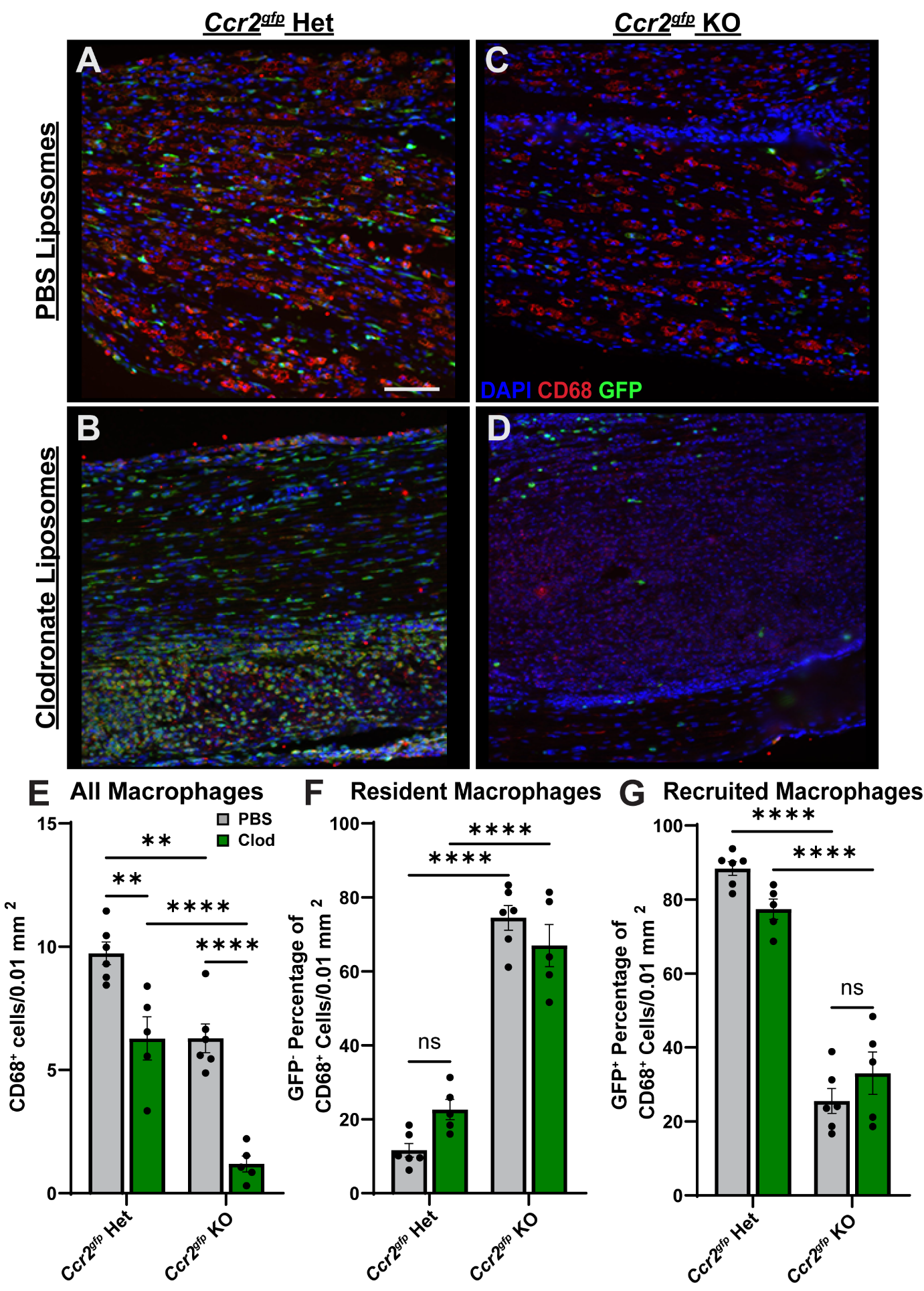


**Figure S5**

A single injection of clodronate liposomes distal to the transection site results in significant macrophage reduction only in *Ccr2^gfp^* KO mice. *Ccr2^gfp^* heterozygous and KO mice received a single injection of clodronate or PBS liposomes (Vehicle) into the sciatic nerve 2 mm distal to the site of a transection injury. Seven days post-injury mice were sacrificed and nerves were stained for CD68, GFP, and DAPI to quantify the macrophage response to injury. **A-D.** Representative images of a nerve area 2 mm distal to the site of injury. **E.** Clodronate liposomes reduced the number of CD68^+^ macrophages in both genotypes. *Ccr2^gfp^* KO mice treated with clodronate liposomes showed a significantly greater reduction in macrophages compared to *Ccr2^gfp^* heterozygous mice. **F.** The percentage of GFP^−^ macrophages was significantly increased in *Ccr2^gfp^* KO mice regardless of liposome treatment. **G.** *Ccr2^gfp^* heterozygous mice had a greater percentage of GFP^+^ macrophages compared to KO mice. N = 5–6 per group. Scale bar = 50 μm. ** *p* < 0.01. **** *p* < 0.0001.


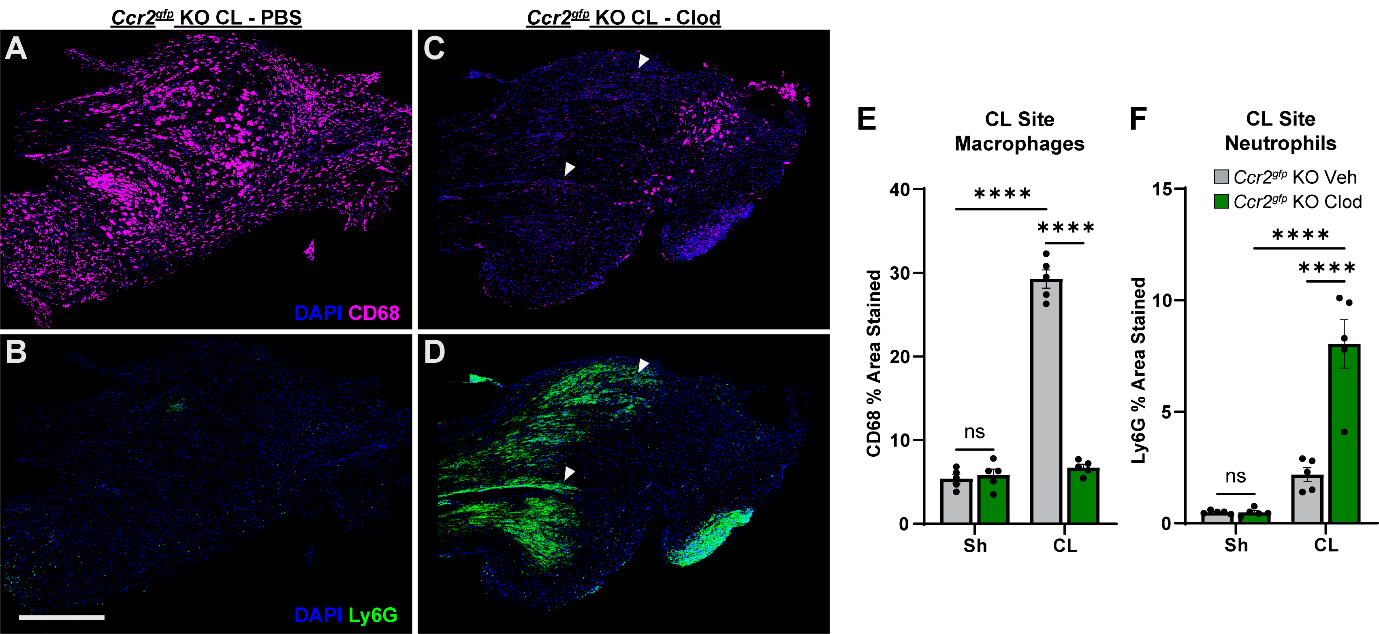


**Figure S6**: Clodronate-depleted *Ccr2^gfp^* KO CL sites display a loss of CD68^+^ macrophages but an increase in Ly6G^+^ neutrophils. The CL site of *Ccr2^gfp^* KO mice treated with PBS or clodronate liposomes was immunostained with DAPI, CD68, and Ly6G. **A-D.** Representative images of vehicle and clodronate liposome treated *Ccr2^gfp^* KO mice stained with DAPI, CD68, and the neutrophil marker Ly6G. Arrowheads indicate areas with colocalization of Ly6G and CD68 staining. **E.** CD68^+^ macrophage staining was ablated in the CL site of clodronate-treated *Ccr2^gfp^* KO mice compared to vehicle-treated mice. **F.** The neutrophil marker, Ly6G, was significantly increased in clodronate-treated CL sites compared to vehicle liposome treatment. Scale bar = 500 μm. N = 5 per group. **** *p* < 0.0001.
